# Engineering a Redox-Responsive Bimolecular Prodrug for Targeted Gastric Cancer Combination Therapy: Design, Synthesis, and In Vivo Evaluation

**DOI:** 10.3390/antiox15091214

**Published:** 2026-09-21

**Authors:** Chao Wang, Yutao Xiu, Yujing Zhang, Jiazhen Xu, Yanhong Wang, Dongming Xing

**Affiliations:** 1 Cancer Institute, The Affiliated Hospital of Qingdao University, Qingdao University, Qingdao 266071, China; wangchao@qdu.edu.cn (C.W.); xiuyutao@qdu.edu.cn (Y.X.); xujiazhen1@qdu.edu.cn (J.X.); 2 Qingdao Cancer Institute, Qingdao University, Qingdao 266071, China; 3 School of Nursing, Qingdao Binhai University, Qingdao 266555, China; 4 School of Life Sciences, Tsinghua University, Beijing 100084, China

**Keywords:** combination chemotherapy, EGFR inhibitor, tubulin inhibitor, redox-responsive prodrug, synergistic effect

## Abstract

Combination chemotherapy often suffers from asynchronous pharmacokinetics and off-target toxicity. To address this, we designed a novel, unreported engineered prodrug—a redox-responsive bimolecular prodrug (CA-4-Gefitinib)—by engineering a disulfide bond as a cleavable linker to covalently conjugate the tubulin inhibitor combretastatin A-4 (CA-4) and the EGFR inhibitor Gefitinib. This molecular-level prodrug design enables tumor-selective drug activation in high-glutathione environments. This prodrug exhibited enhanced cellular uptake and potent antiproliferative activity against SGC-7901 gastric cancer cells, with significantly improved selectivity over normal cells (safety index of 98). Mechanistically, this engineered prodrug disrupted microtubule polymerization, induced G2/M arrest, suppressed ERK signaling, and promoted apoptosis. The prodrug’s pharmacokinetic profile showed prolonged circulation and reduced systemic exposure to free CA-4, indicating favorable biodistribution. In a murine xenograft model, this prodrug demonstrated superior antitumor efficacy and markedly reduced systemic toxicity compared to the combination of free drugs. Our work presents a rational prodrug design strategy—a redox-responsive drug–drug conjugate prodrug—that synchronizes drug delivery, enhances tumor targeting, and maximizes synergistic efficacy, offering a promising platform for advanced cancer combination therapy.

## 1. Introduction

Cancer remains a major cause of mortality worldwide, with conventional monotherapy often yielding limited success due to tumor heterogeneity, drug resistance, metastasis, and recurrence. The complex pathophysiology of tumors, including their adaptive signaling networks, renders single-agent treatments increasingly inadequate. In response, combination chemotherapy has emerged as a cornerstone of modern oncology, leveraging two or more drugs with distinct mechanisms of action to enhance therapeutic efficacy while mitigating resistance and toxicity [1,2,3]. By simultaneously targeting multiple pathways involved in tumor proliferation, survival, and metastasis, this approach can produce synergistic effects that are unattainable with monotherapies [4,5]. Clinically, combinations such as paclitaxel (PTX) with DNA-damaging agents have demonstrated improved outcomes in various malignancies [6,7,8]. Similarly, PTX has been paired with microtubule-targeting agents like combretastatin A-4 (CA-4), P-glycoprotein inhibitors such as tetrandrine, and mTOR inhibitors like everolimus to overcome resistance and broaden antitumor activity [9,10,11]. Notably, epidermal growth factor receptor (EGFR) inhibitors have shown promising synergy with cytotoxic chemotherapies, leading to FDA-approved regimens including erlotinib plus ramucirumab [12,13,14]. These successes underscore the importance of multi-targeted strategies in suppressing tumor adaptation and improving treatment durability.

CA-4, a natural stilbenoid isolated from Combretum caffrum, exhibits potent antitubulin activity by binding to the colchicine site on β-tubulin, thereby inhibiting microtubule polymerization and disrupting mitotic spindle formation [15,16]. This action leads to cell cycle arrest at the G2/M phase and apoptosis in rapidly dividing cells. In addition to its direct cytotoxic effects, CA-4 also acts as a vascular disrupting agent (VDA), inducing rapid shutdown of tumor blood flow through endothelial cell cytoskeletal disruption and thrombotic occlusion [17]. Despite its compelling preclinical profile, the clinical translation of CA-4 and its analogs has been hindered by poor solubility, metabolic instability, and dose-limiting toxicities such as cardiovascular events [18,19]. To address these challenges, advanced formulation strategies—including liposomal encapsulation, polymer conjugates, and prodrug derivatization—have been explored to enhance tumor-specific delivery and reduce systemic exposure [20,21].

Gefitinib, an orally available EGFR tyrosine kinase inhibitor, is widely used in the treatment of non-small cell lung cancer (NSCLC) harboring activating EGFR mutations (e.g., exon 19 deletions and L858R) [22,23]. By blocking ATP binding to the EGFR kinase domain, Gefitinib suppresses downstream signaling pathways such as RAS-RAF-MAPK and PI3K-AKT, inhibiting proliferation and promoting apoptosis in EGFR-driven tumors. However, its efficacy is often compromised by acquired resistance, frequently mediated by the T790M gatekeeper mutation, as well as by dose-dependent toxicities including cutaneous reactions and gastrointestinal disturbances [24,25,26]. To optimize its therapeutic index, ongoing efforts focus on combining Gefitinib with other agents—such as chemotherapy, angiogenesis inhibitors, or immune modulators—to enhance response and delay resistance [27].

In recent years, molecular hybridization and prodrug strategies have gained traction as rational approaches to improve combination chemotherapy [28,29]. Bimolecular prodrugs, in particular, allow for coordinated delivery of two bioactive agents via a cleavable linker, enabling tumor-selective activation and reduced off-target toxicity [30,31]. Among various stimulus-responsive linkers, disulfide bonds have been extensively utilized due to their high stability in circulation and rapid cleavage in the reductive tumor microenvironment, where glutathione (GSH) concentrations are significantly elevated compared to normal tissues [32,33,34]. This redox-dependent release mechanism offers spatial control over drug activation, maximizing intracellular drug concentrations at the target site while minimizing systemic side effects [35].

**Figure 1:**
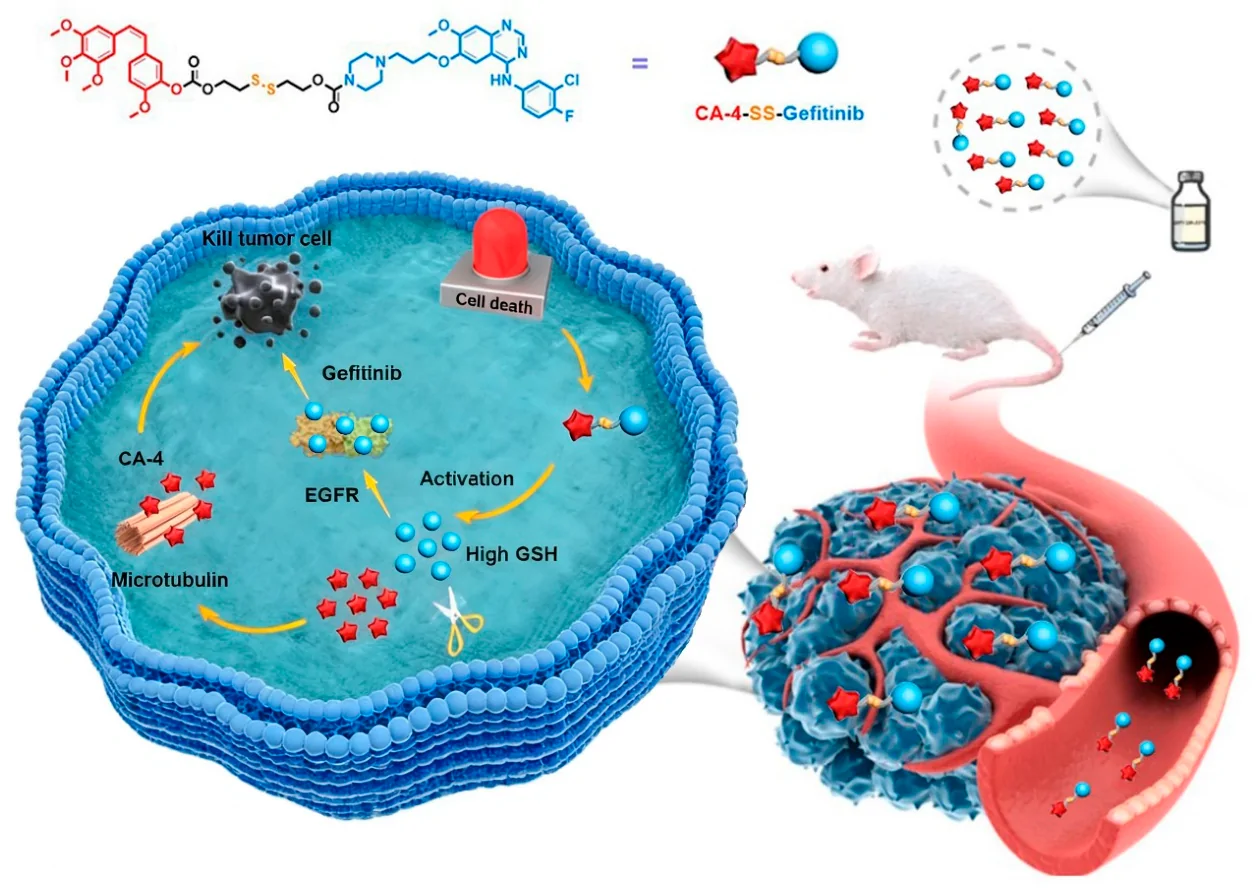
Schematic illustration of bimolecular prodrug in the treatment of gastric cancer.

In our preliminary screening, we identified a strong synergistic interaction between CA-4 and Gefitinib in gastric adenocarcinoma models (Figure S1), suggesting a promising therapeutic avenue. Despite their complementary mechanisms—wherein CA-4 induces microtubule disruption and mitotic arrest, while Gefitinib suppresses EGFR-driven survival and proliferation pathways—their conventional co-administration remains challenged by divergent pharmacokinetics, inadequate tumor-specific accumulation, and potential additive systemic toxicities. To overcome these limitations, we designed and synthesized a novel, previously unreported redox-responsive CA-4–Gefitinib conjugate covalently linked via a disulfide bond (Figure 1). This bifunctional prodrug was thoroughly characterized using HPLC-MS, ^1^H NMR, and ^13^C NMR, and its performance was systematically evaluated across multiple dimensions: redox-sensitive drug release under simulated physiological conditions, cellular uptake efficiency, in vitro antiproliferative potency, and mechanistic effects on microtubule dynamics, cell cycle progression, and apoptosis induction. Additionally, we assessed its anti-migratory and anti-angiogenic functions, pharmacokinetic profile, tumor-targeting capability, in vivo antitumor efficacy in xenograft models, and overall systemic safety. By integrating the dual actions of vascular disruption and mitotic arrest from CA-4 with the signaling blockade of Gefitinib into a single tumor-microenvironment-activated molecule, this bimolecular prodrug platform offers a targeted, synergistic, and safety-enhanced approach for the treatment of gastric adenocarcinoma.

## 2. Materials and Methods

### 2.1. Materials

We utilized reagents and chemicals that are commercially available without undergoing further purification, unless otherwise stated. The ^13^C NMR and ^1^H NMR spectra were acquired using an AVANCE spectrometer (Bruker, Karlsruhe, Germany) in CDCl_3_ at 126 MHz or 500 MHz. Mass spectrometry data were collected using a Q-TOF 6530 mass spectrometer (Agilent, Singapore). The separation of compounds was carried out using 100–300 mesh silica gel in column chromatography. For accurate quantification, the UPLC system (Shimadzu, Kyoto, Japan) connected with a triple quadrupole QTRAP 5500 mass spectrometer (Redwood City, CA, USA) was utilized. Chromatographic separation was performed using a C18 column (Exsil Mono, 3 μm, 2 mm × 50 mm) from Dr. Maisch GmbH. The temperature settings for the autosampler and column were maintained at a constant level of 4 °C and 35 °C, respectively.

### 2.2. General Synthetic Procedure for the Synthesis of 4-((3-Chloro-4-fluorophenyl)amino)-7-methoxyquinazolin-6-ol (***5***)

Intermediate **5** was synthesized in accordance with the reported procedures [36].

### 2.3. General Synthetic Procedure for N-(3-Chloro-4-fluorophenyl)-7-methoxy-6-(3-(piperazin-1-yl)propoxy)quinazolin-4-amine (***8***)

Tert-butyl 4-(3-bromopropyl)piperazine-1-carboxylate (**6**, 1.0 mmol, 306 mg) was added to a suspension of potassium carbonate (3.0 mmol, 414 mg) and **5** (1.0 mmol, 445 mg) in 15 mL *N*,*N*-dimethylformamide. Following stirring at 85 °C for 12 h, water was introduced and the mixed reaction solution was extracted with dichloromethane (15 mL × 3), dried over Na_2_SO_4_, concentrated, purified, and eluted with MeOH/DCM (*v*/*v* 50:1) to obtain intermediate **7**. Intermediate **7** was dissolved in TFA (7 mL) and DCM (7 mL). The resultant mixture was stirred for 0.5 h. Subsequently, it was concentrated and the pH was adjusted to approximately 9 through the addition of saturated NaHCO_3_ solution. After that, the crude product was eluted with MeOH/DCM (*v*/*v* 20:1) to obtain the key intermediate **8** [37].

### 2.4. General Synthetic Procedure for 2-((2-Hydroxyethyl)disulfanyl)ethyl 4-(3-((4-((3-chloro-4-fluorophenyl)amino)-7-methoxyquinazolin-6-yl)oxy)propyl)piperazine-1-carboxylate (***10***)

A solution of 2-((2-hydroxyethyl)dithio)ethyl (4-nitrophenyl) carbonate (**9**, 0.68 mmol, 218 mg) in 10 mL of THF was added to compound **8** (1 mmol, 457 mg) and stirred at rt. for 4 h. Subsequently, the solvent was removed by means of rotary evaporation. The resultant residue was dissolved in 15 mL of dichloromethane, washed with saturated NaHCO_3_ solution (25 mL × 3), and brine (20 mL × 3), and then dried over anhydrous MgSO_4_ overnight. Upon the removal of the solvent, purification was carried out using recrystallization or column chromatography to give compound **10** [38]. MS (ESI) *m*/*z* 625.2 [M + H]^+^.

### 2.5. General Synthetic Procedure for (Z)-2-((2-(((2-Methoxy-5-(3,4,5-trimethoxystyryl)phenoxy)carbonyl)oxy)ethyl)disulfanyl)ethyl 4-(3-((4-((3-chloro-4-fluorophenyl)amino)-7-methoxyquinazolin-6-yl)oxy)propyl)piperazine-1-carboxylate (***12***, CA-4-Gefitinib bimolecular prodrug)

CA-4 (**11**, 1 mmol, 316 mg), triphosgene (0.3 mmol, 99 mg), and DMAP (1 mmol, 122 mg) were dissolved in a solution of anhydrous dichloromethane under argon, heated, and stirred for 2.5 h, after which compound **10** (1 mmol, 626 mg) and DIPEA (0.5 mmol, 64.6 mg) were added. After stirring, the reaction solution was allowed to react for 24 h at 30 °C. After completion of the reaction, the reaction solution was washed with 0.1 M HCl solution and extracted with dichloromethane (30 mL × 3). The extracted organic solution was washed with saturated brine and dried with MgSO_4_, and then purified by silica gel column chromatography (n-Hexane/EtOAc = 1/2)) to give a white solid product, compound **12** [39]. M.p. 103.2–105.1 °C; ^1^H NMR (CDCl_3_, 400 MHz) δ 8.63 (s, 1H), 7.86 (dd, *J* = 6.6, 2.7 Hz, 1H), 7.64 (s, 1H), 7.53 (ddd, *J* = 8.9, 4.1, 2.7 Hz, 1H), 7.22 (s, 1H), 7.17 (s, 1H), 7.15–7.05 (m, 3H), 6.82 (d, *J* = 8.4 Hz, 1H), 6.46 (s, 2H), 6.42 (s, 2H), 4.44 (t, *J* = 6.8 Hz, 2H), 4.33 (t, *J* = 6.3 Hz, 2H), 4.10 (t, *J* = 6.5 Hz, 2H), 3.96 (s, 3H), 3.81 (s, 3H), 3.80 (s, 3H), 3.67 (s, 6H), 3.48 (d, *J* = 5.5 Hz, 4H), 2.96 (dt, *J* = 12.5, 6.5 Hz, 4H), 2.54 (t, *J* = 7.0 Hz, 2H), 2.42 (d, *J* = 5.3 Hz, 4H), 2.05 (p, *J* = 6.8 Hz, 2H); ^13^C NMR (101 MHz, CDCl_3_) δ 169.92 (C), 156.32 (C), 155.26 (C), 155.22 (C), 154.95 (C), 153.49 (C), 153.12 (C), 152.99 (2C, C), 150.24 (C), 149.08 (C), 147.57 (C), 139.68 (C), 137.18 (CH), 136.70 (C), 135.53 (C), 132.42 (CH), 130.19 (CH), 129.86 (CH), 128.44 (CH), 128.15 (CH), 124.23 (CH), 122.77 (C), 121.87 (CH), 121.80 (CH), 116.75 (CH), 116.54 (CH), 112.24 (CH), 109.05 (CH), 107.94 (CH), 105.91 (2C, CH), 100.94 (CH), 67.53 (CH_2_), 66.55 (CH_2_), 63.17 (CH_2_), 61.03 (CH_2_), 56.30 (CH_3_), 56.10 (CH_3_), 55.97 (2C, CH_3_), 54.93 (CH_2_), 52.92 (CH_2_), 37.94 (CH_2_), 36.80 (CH_2_), 26.32 (CH_2_). HRMS calcd for C_16_H_16_BrN_2_O_3_ [M + H]^+^ 968.2777, found 968.2796.

### 2.6. GSH-Activatable Response Bimolecular Prodrug

At 0 h, 20 µL of a 2 mM stock solution of the prodrug was added to 980 µL of glutathione solution (20 mM, prepared in the mobile phase of 20 mM ammonium acetate aqueous solution). The mixture was thoroughly mixed, and then 100 µL of this solution was immediately transferred and combined with 100 µL of blank diluent. After vortexing and mixing well, the sample was centrifuged at 1200 rpm for 5 min and subsequently injected for analysis. At 2 h, 20 µL of the 2 mM prodrug solution was added to 980 µL of glutathione solution (20 mM, prepared in 20 mM NH_4_OAc aqueous solution as the mobile phase). The mixture was mixed well and incubated with shaking at 37 °C for 2 h. Then, 100 µL of the reaction mixture was taken and combined with 100 µL of blank diluent. After vortexing and thorough mixing, the sample was centrifuged at 1200 rpm for 5 min and then injected for analysis [40].

### 2.7. In Vitro Release of CA-4 and Gefitinib Analog from Bimolecular Prodrug

A 2 mM stock solution of the prodrug was added to GSH solution (20 mM). The mixture was thoroughly mixed and incubated with shaking at 37 °C. At 0.5, 1, 2, 4, 8, 12 and 24 h after the start of the reaction, 100 µL aliquots of the reaction solution were collected and mixed with blank diluent to terminate the reaction. A certain amount of methanol was subsequently added to each sample for dilution. After vortexing to ensure thorough mixing, the samples were centrifuged at 1200 rpm for 5 min and then injected for analysis. A parallel control group was prepared using a GSH-free solution under the same conditions [40].

### 2.8. Cell Culture

Different human cancer cell lines (HCT-116, SGC-7901, HepG2, MCF-7, A549, HCC827, and NCI-H1975), and the human normal cell line HUVEC were acquired from the Chinese Academy of Sciences Cell Bank. HCT-116, SGC-7901, HepG2, and MCF-7 cell lines were cultured in modified BasalMedia DMEM medium (L110KJ, (Shanghai, China) supplemented with PWL062 penicillin–streptomycin solution and PWL001 fetal bovine serum (Meilunbio, Dalian, China). HCC827 and NCI-H1975 cell lines were maintained in BasalMedia L210KJ medium (RPMI-1640). A549 cells were cultured in Ham’s F-12K medium (L450KJ). HUVECs were cultured in a complete culture medium for HUVECs (HUVEC-90011, Oricell, Guangzhou, China).

### 2.9. In Vitro Anti-Proliferative Activity

The cytotoxicity of Gefitinib, CA-4, CA-4 + Gefitinib, or CA-4-Gefitinib bimolecular prodrug in different cells was detected by CCK-8 assay. In brief, 4 × 10^3^/well cells were seeded into 96-well plates and then exposed to CA-4 (0–500 nM), Gefitinib (0–50 μM), CA-4 + Gefitinib (0–500 nM), or CA-4-Gefitinib bimolecular prodrug (0–1.5 μM) for 72 h. Cell viability was then determined using the Melunbio MA0218 CCK-8 kit. GraphPad software version 8.0 was used to determine IC_50_ values [41].

### 2.10. Drug Interaction Analysis

SGC-7901 cells (4 × 10^3^/well) were treated with CA-4 (0–26 nM) or Gefitinib (0–44 μM) combined or alone. From the IC_50_ values calculated from the cytotoxicity assays described above, concentration gradients for CA-4 or Gefitinib were determined. After 72 h of incubation, cell viability was assessed by the CCK8 assay. Finally, the drug–drug interaction between CA-4 and Gefitinib was predicted using the online SynergyFinder software (version 2.0). Synergy scores were determined by the Zero Interaction Potency (ZIP) calculation method using ‘vigour readings’. Synergy scores (ZIP) < −10 were considered to be antagonistic and scores ≥ −10 were considered to be additive synergistic [42].

### 2.11. Cellular Uptake CA-4-Gefitinib Bimolecular Prodrug in SGC-7901 Cells

A cell lysis assay was used to determine the cellular uptake of the CA-4-Gefitinib bimolecular prodrug. The determination of the cellular uptake of the CA-4-Gefitinib bimolecular prodrug by the cells was carried out through UPLC-MS analysis [40].

### 2.12. Colony Formation Assay

For the detection of cell proliferation following diverse treatments, either involving CA-4 (7 nM), Gefitinib (22 μM), CA-4 + Gefitinib (7 nM), or CA-4-Gefitinib bimolecular prodrug (7 nM), a colony formation assay was carried out. The number of colonies formed was calculated using the USA National Institutes of Health ImageJ software (version 1.54f) [43].

### 2.13. Immunofluorescence Staining Analysis

Microtubule-associated tubulin was detected by immunostaining after different treatments. SGC-7901 cells were plated at 2 × 10^4^ per well in 24-well plates and treated with CA-4 (7 nM), CA-4 + Gefitinib (7 nM), or CA-4-Gefitinib bimolecular prodrug (7 nM) for 24 h. Cells were then fixed with P1110 paraformaldehyde for 0.5 h, permeabilised with T8200 Triton X-100 (Solarbio, Beijing, China) for 5 min, and fixed with Solarbio A8020 bovine serum albumin (BSA, A8020, Solarbio) for 0.5 h. Then the cells were fixed with paraformaldehyde (P1110, Solarbio, Beijing, China) for 30 min, permeabilized with 0.1% (*v*/*v*) Triton X-100 (T8200, Solarbio, Beijing, China) for 5 min, and blocked with 5% bovine serum albumin (BSA, A8020, Solarbio, Beijing, China) for 30 min. After incubation with the primary α-tubulin antibodies (A6830, Abclonal, Wuhan, Hubei, China) at 4 °C for 12 h, the cells were preincubated with Abclonal AS011 FITC-conjugated anti-IgG for 1 h, after which the nuclei were counterstained with Beyotime C1005 DAPI. Images were acquired with a Germany ZEISS LSM 880 laser (Carl Zeiss Microscopy GmbH, Jena, Germany) scanning confocal microscope [41].

### 2.14. Cell Cycle Analysis

Cell cycle distribution was ascertained by employing the Biotronik C1052 Cell Cycle Assay Kit (Beyotime, Shanghai, China) in accordance with the manufacturer’s instructions. In short, after undergoing various treatments, either with CA-4 (7 nM), Gefitinib (22 μM), CA-4 + Gefitinib (7 nM), or CA-4-Gefitinib bimolecular prodrug (7 nM) for 24 h, SGC-7901 cells were ethanol-fixed, then processed in the dark at 37 °C with PI solution and finally analyzed by flow Beckman Coulter CytoFlex cytometry. Analysis was performed using USA Verity Software House ModFit LT software (version 4.1) [41].

### 2.15. Cell Apoptosis Analysis

The apoptosis rate was analyzed by employing the Yeasen Annexin V-FITC/PI Apoptosis Detection Kit (Beyotime, Shanghai, China) in line with the manufacturer’s instructions. Summarily, after undergoing various treatments, either with CA-4 (7 nM), Gefitinib (22 μM), CA-4 + Gefitinib (7 nM), or CA-4-Gefitinib bimolecular prodrug (7 nM) for 48 h, SGC-7901 cells were lysed with EDTA-free trypsin (MA0234, Meilun, Dalian, China), followed by incubation with Annexin V-FITC and PI (Yeasen, Shanghai, China) for 15 min in the dark and flow cytometric analysis [41].

### 2.16. Wound-Healing Assay

Cell migration was evaluated by means of a wound-healing assay. Briefly, SGC-7901 cells were inoculated into a 6-well plate, grown to confluence, and then a line was scraped across the cell monolayer using a 200 μL sterile pipette tip. After scraping off the cell debris, the cell monolayer was incubated with CA-4 (3.5 nM), Gefitinib (5.5 μM), CA-4 + Gefitinib (3.5 nM), or CA-4-Gefitinib bimolecular prodrug (3.5 nM) for 24 h. Images were taken under a Nikon ECLIPSE Ts2 inverted microscope after scraping. Wound closure rates were quantified using ImageJ software (version 1.54f) [44,45].

### 2.17. Tube Formation Assay

Tube formation tests are used to assess the effect of drugs on microvessel formation. Matrigel (356234, Corning Incorporated, Tewksbury, MA, USA) was melted at 4 °C prior to the experiment. Add 50 μL of Matrigel to pre-cooled 96-well plates and incubate at 37 °C for 60 min. HUVECs (3 × 10^4^/well) were added to each well, and the drugs containing CA-4 (7 nM), Gefitinib (22 μM), CA-4 + Gefitinib (7 nM), or CA-4-Gefitinib bimolecular prodrug (7 nM) were then placed in 96-well plates. Following an incubation period of 3 h at 37 °C, the tubular structures of HUVECs were visualized by an inverted microscope [45].

### 2.18. Pharmacokinetic Studies

In the pharmacokinetic study, nine rats (Sprague-Dawley) were randomly assigned to three groups: the CA-4-Gefitinib bimolecular prodrug group, the CA-4 group, and the Gefitinib group. Group I rats received a single dose of CA-4-Gefitinib bimolecular prodrug (equivalent to 3.7 mg/kg CA-4 and 5.3 mg/kg Gefitinib). Group II rats were intravenously administered 3.7 mg/kg CA-4. Group III rats received an intravenous administration of 5.3 mg/kg Gefitinib. Blood samples were obtained from the rats by retro-orbital puncture between 5 min and 24 h after injection. Validated methods were used to prepare and assay the samples. For the calculation of pharmacokinetic parameters, non-compartmental model analysis was performed using Phoenix WinNonlin 7.0 [46,47].

### 2.19. In Vivo Antitumour Activity Assay

The in vivo antitumor effects of the CA-4-Gefitinib bimolecular prodrug were evaluated in an SGC-7901 cell xenograft model in mice. Male NOD-SCID IL2Rγ-null (NSG) mice were obtained from Beijing Viewsolid Biotechnology Company. The right axillary subcutaneous tissue was injected with SGC-7901 cells. Tumor growth was assessed as tumor volume (mm^3^), calculated as (width^2^ × length)/2. Saline was used for the control group. The CA-4 + Gefitinib group was administered 21.2 mg/kg Gefitinib and 15.0 mg/kg CA-4. The CA-4-Gefitinib bimolecular prodrug group was administrated 46.0 mg/kg. The drug was administered via the tail vein every 2 days. Tumor size and mouse body weight were recorded every two days. Two weeks after the first administration of the drug, the mice were euthanized, and the tumors were excised and weighed [41].

### 2.20. Evaluation of In Vivo Safety

To evaluate the safety of the CA-4-Gefitinib bimolecular prodrug, vehicle or CA-4-Gefitinib bimolecular prodrug (46.0 mg/kg) was intravenously administered to the ICR mice every two days (*n* = 3). Ten days after the initiation of treatment, blood samples were taken for blood chemistry and hematology analyses. In addition, the major organs (liver, heart, lung, kidney, and spleen) were collected and fixed in paraformaldehyde. The organs were immersed in paraffin wax, sectioned, and stained with H&E for histological assessment. Finally, a Panoramic Tissue Cell Scanning Analyzer (Pannoramic MIDI, 3DHISTECH, Hungary) was used to scan the slides [41].

### 2.21. Determination of Drug Content in Tumor Tissue

In order to further determine the inhibitory effect of the CA-4-Gefitinib bimolecular prodrug on mouse tumors in vivo, after 2 weeks of administration, tumor tissues were removed from the mice in the positive control group and the mice in the CA-4-Gefitinib bimolecular prodrug group, respectively, and the tumor tissues of the groups were analyzed by UPLC-MS to determine the levels of the active ingredients CA-4 and Gefitinib [46,47].

## 3. Results

### 3.1. Rational Design and Synthesis of Bimolecular Prodrug

**Scheme 1:**
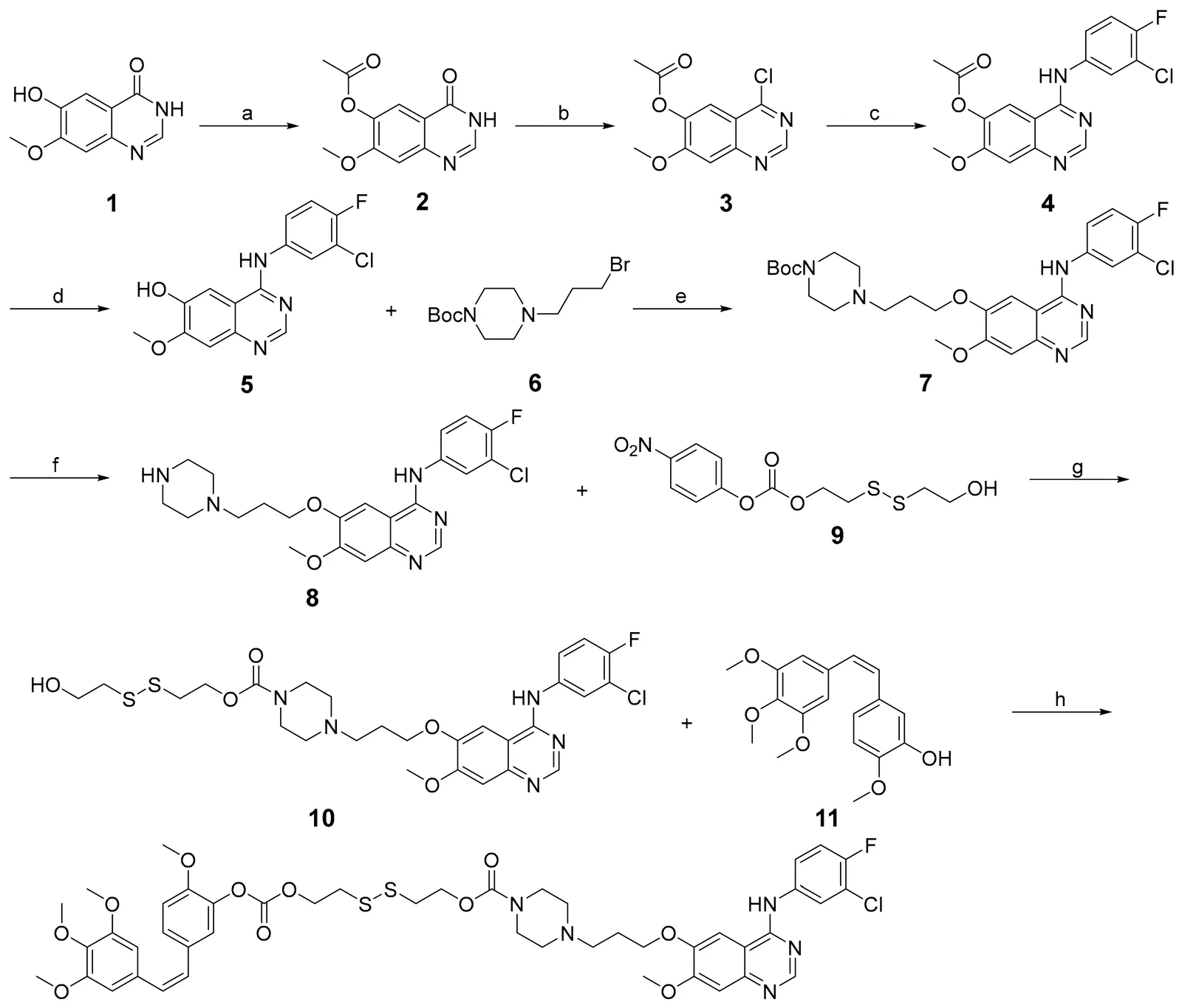
Reagents and conditions: (a) (CH_3_CO)_2_O, pyridine, reflux; (b) DMF, SOCl_2_, reflux; (c) 3-chloro-4-fluoroanilin, CH_3_CN, reflux; (d) NH_3_⋅H_2_O, CH_3_OH, reflux; (e) DMF, K_2_CO_3_, 80 °C; (f) DCM, TFA, rt; (g) DCM, rt; (h) DMAP, triphosgene, DIPEA, DCM, rt.

The synthetic route for the redox-sensitive CA-4-Gefitinib bimolecular prodrug is delineated in Scheme 1. The design rationale centered on covalently tethering the microtubule-targeting agent CA-4 to the EGFR inhibitor Gefitinib via a disulfide bond, which serves as a cleavable linker responsive to the reductive tumor microenvironment. This strategy aims to ensure coordinated delivery of both therapeutic agents to the tumor site, with subsequent intracellular release of the active drugs upon glutathione-mediated reduction.

The synthesis commenced with the preparation of the key Gefitinib analog intermediate (**8**). Briefly, compound **5** was synthesized according to a previously reported method [36]. Subsequently, a nucleophilic substitution reaction between **5** and tert-butyl 4-(3-bromopropyl)piperazine-1-carboxylate (**6**) afforded compound **7**. Deprotection of the N-Boc group in **7** using trifluoroacetic acid yielded the Gefitinib precursor **8** [37]. In parallel, the disulfide-containing linker intermediate (**10**) was constructed. Commercially available 2,2′-dithiodiethanol was reacted with 4-nitrophenyl chloroformate in anhydrous tetrahydrofuran in the presence of triethylamine, yielding the activated carbonate derivative **9**. This intermediate was then coupled with the amino group of compound **8** to furnish the Gefitinib-disulfide conjugate **10** [38]. For the final conjugation, CA-4 (**11**) was first activated by reaction with triphosgene in the presence of *N*,*N*-diisopropylethylamine (DIPEA) and a catalytic amount of 4-dimethylaminopyridine (DMAP). The resulting CA-4-derived chloroformate intermediate was then reacted with the hydroxyl group of the disulfide-linked Gefitinib analog (**10**), culminating in the formation of the target CA-4-Gefitinib bimolecular prodrug (**12**) in moderate yield [39]. The structure of the final prodrug was unequivocally confirmed by comprehensive spectroscopic analysis, including ^1^H NMR, ^13^C NMR, and high-resolution mass spectrometry (HRMS), the data of which were consistent with the proposed structure (Figures S4–S6).

### 3.2. In Vitro Release of CA-4 and Gefitinib from Bimolecular Prodrug

**Figure 2:**
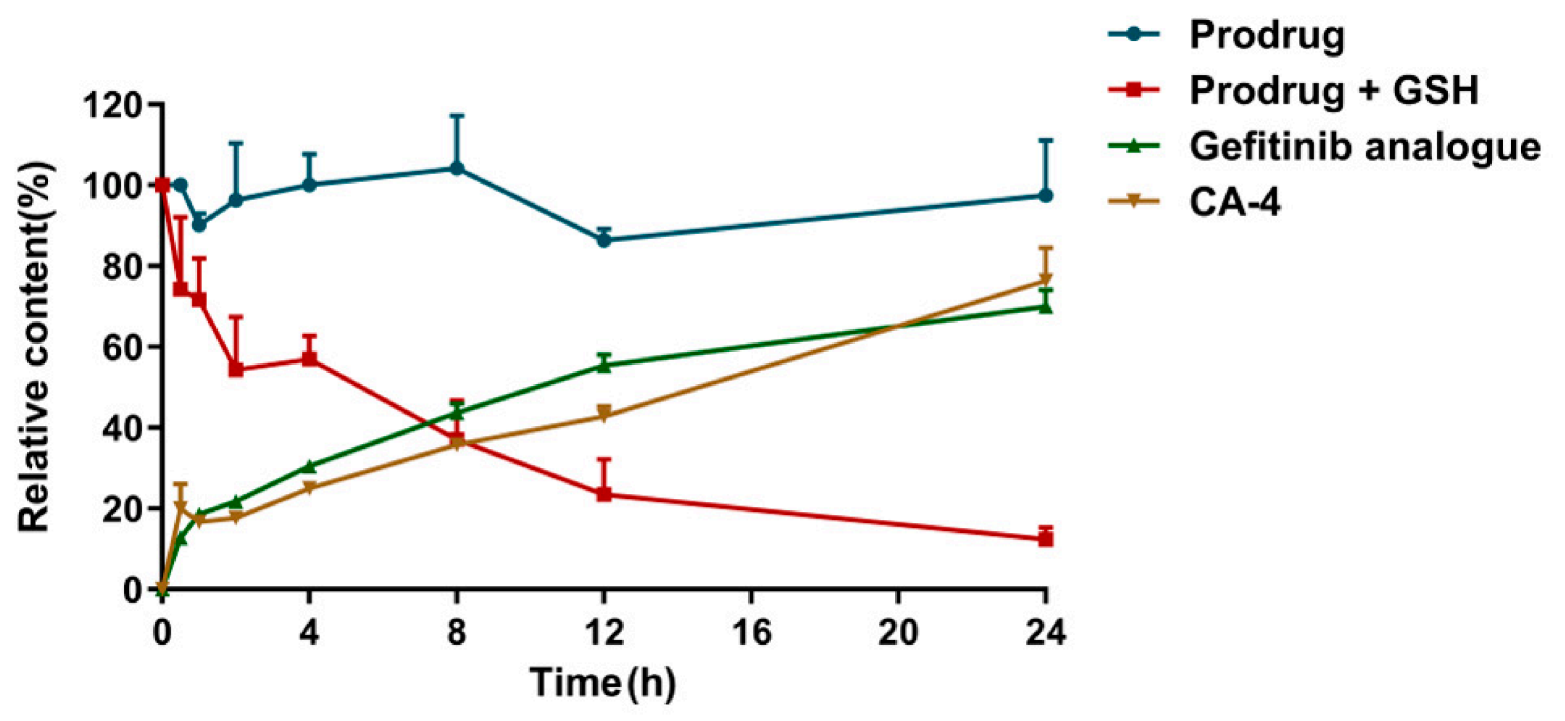
In vitro release of CA-4 and Gefitinib analog from bimolecular prodrug.

To validate the redox-sensitive release mechanism of the CA-4-Gefitinib bimolecular prodrug, we investigated its drug release behavior under simulated physiological and reductive conditions. The prodrug (2 mM) was incubated in phosphate-buffered saline (PBS, pH 7.4) in both the absence and presence of GSH (20 mM) to mimic the normal extracellular and intracellular tumor microenvironmental conditions, respectively. As illustrated in Figure 2, the release of free CA-4 and the Gefitinib analog from the prodrug conjugate was negligible over 24 h in PBS without GSH, indicating high stability under physiological conditions. In stark contrast, upon exposure to 20 mM GSH, a rapid and substantial release was observed, with over 40% of both drugs liberated within the first 12 h. The release profile continued to increase, reaching nearly 80% after 24 h of incubation. These results clearly demonstrate that the disulfide linker within the prodrug structure is highly stable under low-reducing conditions but undergoes efficient cleavage in a high-GSH environment, confirming the redox-responsive drug release characteristic.

The mechanism of GSH-triggered release was further elucidated using reverse-phase UPLC-MS analysis. As shown in Figure S2, the intact CA-4-Gefitinib prodrug exhibited a single peak at a retention time of 28.5 min, which corresponded to the protonated molecular ion [M + H]^+^ at * *m*/*z* * 968.2733 in the HRMS spectrum. Following a 2 h incubation with GSH, this peak significantly diminished. Concurrently, two new prominent peaks emerged at retention times of 6.35 min and 28.6 min, which were unambiguously assigned to the released free CA-4 and Gefitinib analog, respectively, based on their HRMS signals at * *m*/*z* * 317.1374 [M + H]^+^ for CA-4 and * *m*/*z* * 446.17328 [M + H]^+^ for the Gefitinib analog. This chromatographic and mass spectrometric evidence confirms that the prodrug undergoes reductive cleavage at the disulfide bond, likely via a thiol–disulfide exchange reaction facilitated by GSH, leading to the simultaneous release of the two active parent drugs. The efficient cleavage and release kinetics support the design rationale that the prodrug can remain stable during systemic circulation but rapidly liberate the active agents upon entry into the reducing cytoplasm of tumor cells.

### 3.3. The Bimolecular Prodrug Enhances Anticancer Selectivity

The cytotoxic activity of the CA-4-Gefitinib bimolecular prodrug was systematically evaluated against a panel of human cancer cell lines—including SGC-7901 (gastric adenocarcinoma), MCF-7 (breast cancer), HepG2 (hepatocellular carcinoma), HCT-116 (colorectal carcinoma), and A549 (non-small cell lung cancer)—along with corresponding normal cells using the CCK-8 assay. As summarized in Table 1, the prodrug exhibited potent and broad-spectrum antiproliferative effects, with IC_50_ values ranging from 7.17 ± 0.65 nM to 30.89 ± 0.93 nM across the tested cancer lines. Notably, the prodrug demonstrated superior potency compared to free CA-4, free Gefitinib, and their physical combination, highlighting the advantage of the covalently linked prodrug approach in enhancing cytotoxic efficacy.

A key finding was the significantly improved selectivity profile of the prodrug. While CA-4 alone and the CA-4/Gefitinib combination showed considerable toxicity toward non-malignant human umbilical vein endothelial cells (HUVECs), the CA-4-Gefitinib prodrug exhibited markedly reduced cytotoxicity in these normal cells. This enhanced safety profile was quantitatively reflected in the safety index (SI), calculated as the ratio of IC_50_ in HUVECs to IC_50_ in SGC-7901 cells. The prodrug achieved an SI value of 98, substantially higher than those of CA-4 (**15**), Gefitinib (**1**), and their combination (**12**). We attribute this improved selectivity to the redox-sensitive design of the prodrug, which remains stable under normal physiological conditions but undergoes rapid cleavage in the high-GSH environment characteristic of tumor cells, thereby minimizing off-target effects in healthy tissues.

Importantly, the prodrug showed exceptional activity against SGC-7901 gastric adenocarcinoma cells, the model used for subsequent in vitro and in vivo studies. To the best of our knowledge, this is the first report detailing the potent and selective anticancer effects of a redox-responsive CA-4-Gefitinib conjugate in this clinically relevant cell line. These results collectively underscore the potential of the bimolecular prodrug strategy to enhance therapeutic efficacy while reducing systemic toxicity, providing a strong rationale for its further development as a targeted combination therapy.

### 3.4. Cellular Uptake and Intracellular Activation of the Bimolecular Prodrug in SGC-7901 Cells

**Figure 3:**
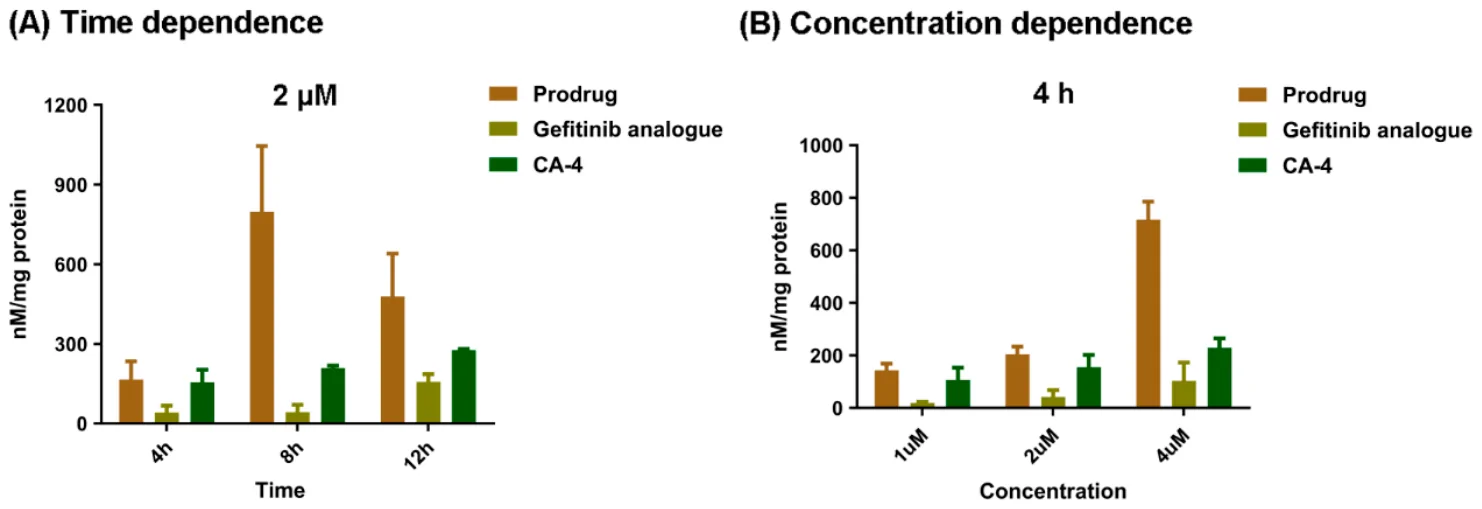
Intracellular uptake of bimolecular prodrug on tumor cell.

To investigate the cellular uptake efficiency and glutathione-triggered activation of the CA-4-Gefitinib bimolecular prodrug, we quantitatively analyzed the intracellular concentrations of the prodrug and its released active components in SGC-7901 gastric cancer cells using UPLC-MS. As shown in Figure 3A, when SGC-7901 cells were treated with 2 μM prodrug, all three analytes—the intact prodrug, free CA-4, and the Gefitinib analog—were detected intracellularly within 4 h. The intracellular level of the intact prodrug reached a maximum at 8 h, after which it gradually decreased by 12 h. Conversely, the concentrations of free CA-4 and the Gefitinib analog increased correspondingly after the 8 h time point, indicating progressive intracellular cleavage of the prodrug.

To further examine the concentration-dependent uptake and activation, SGC-7901 cells were exposed to increasing concentrations of the prodrug (1, 2, and 4 μM) for 4 h. As summarized in Figure 3B, even at the lowest concentration of 1 μM, significant intracellular levels of both the prodrug and its active metabolites were detected. Moreover, the intracellular amounts of all three analytes rose in a dose-dependent manner, confirming that cellular uptake and subsequent activation are positively correlated with extracellular prodrug concentration.

These results collectively demonstrate that the CA-4-Gefitinib bimolecular prodrug is efficiently internalized by SGC-7901 cells and undergoes rapid intracellular reduction, leading to the release of active CA-4 and Gefitinib analog. The time- and concentration-dependent trends support the proposed mechanism of redox-responsive activation, highlighting the potential of this prodrug strategy for tumor-specific drug delivery.

### 3.5. The Bimolecular Prodrug Potently Inhibits SGC-7901 Cell Proliferation

**Figure 4:**
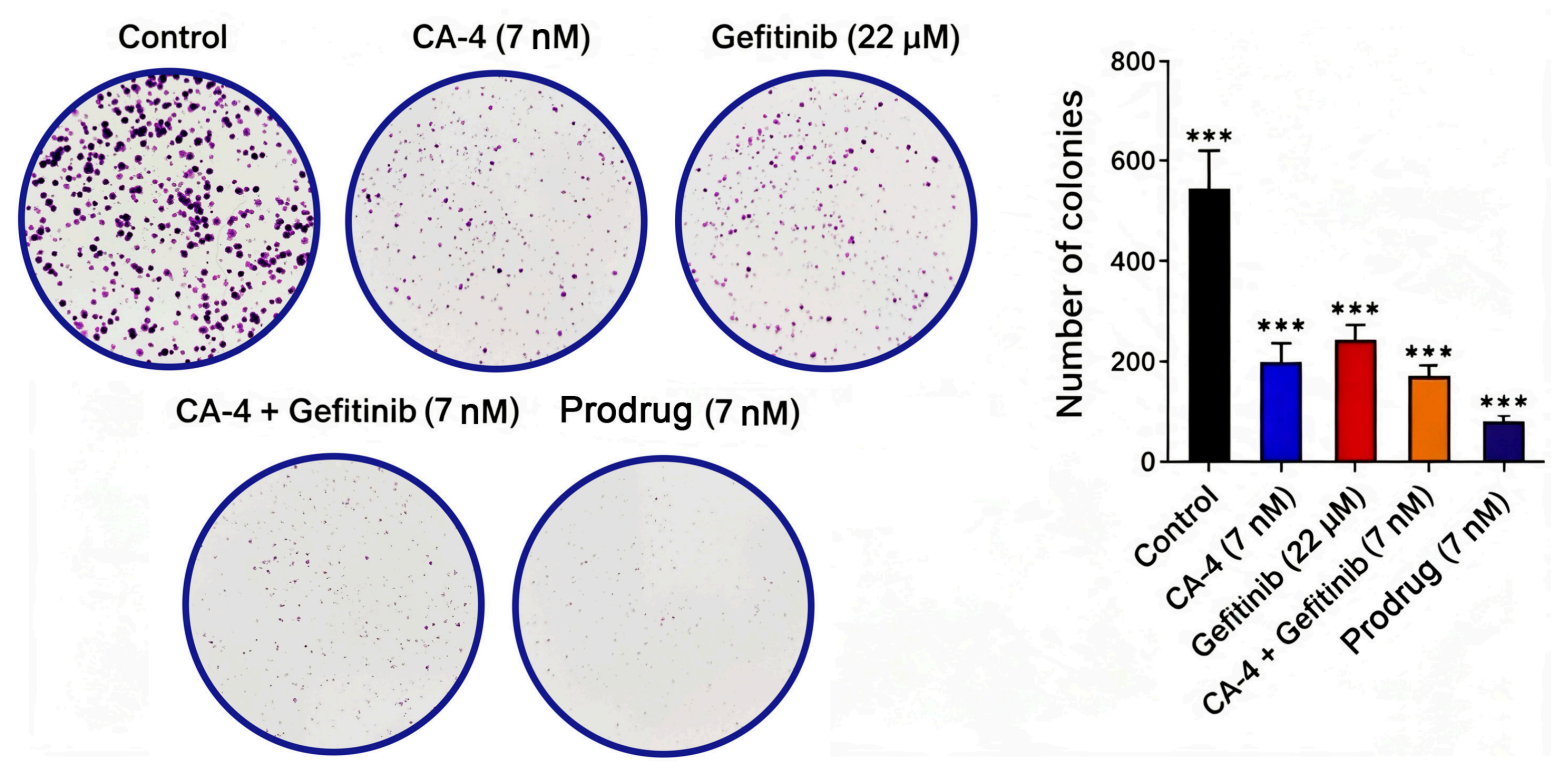
Bimolecular prodrug inhibited cell proliferation in SGC-7901 cells. Under different treatments with either Gefitinib, CA-4, CA-4 + Gefitinib, or bimolecular prodrug, colony formation capacity was determined by a colony formation assay in SGC-7901 cells. Error bars, SEM. *** *p* < 0.001.

To evaluate the long-term proliferative capacity of gastric cancer cells following drug exposure, we performed a colony formation assay in SGC-7901 cells treated with CA-4 (7 nM), Gefitinib (22 μM), the physical combination of CA-4 and Gefitinib (7 nM each), or the CA-4-Gefitinib bimolecular prodrug at 7 nM. As shown in Figure 4, all treatment groups exhibited significantly reduced colony numbers relative to the untreated control, confirming the growth-inhibitory activity of each regimen.

Among the single-agent treatments, both CA-4 and Gefitinib markedly suppressed colony formation, with CA-4 demonstrating a more pronounced effect in line with its potent cytotoxic profile. Co-administration of the two free drugs yielded a stronger inhibitory outcome than either monotherapy, corroborating the synergistic interaction identified in our earlier screening. Strikingly, the bimolecular prodrug at the same 7 nM concentration achieved the most robust suppression of colony formation among all groups, with a statistically significant reduction compared not only to each single agent but also to the free drug combination.

These results demonstrate that the redox-responsive prodrug not only preserves the synergistic anti-proliferative activity of the CA-4/Gefitinib pair but also substantially amplifies its potency against SGC-7901 cells. The superior performance of the prodrug is likely attributable to its ability to deliver both active agents simultaneously and intracellularly in a coordinated manner, thereby maximizing the cooperative inhibitory effect on sustained clonogenic growth. Collectively, these findings underscore the therapeutic advantage of the bimolecular prodrug strategy in overcoming the limitations of conventional drug combinations.

### 3.6. The Bimolecular Prodrug Disrupts the Microtubule Network in SGC-7901 Cells

CA-4 is a well-established microtubule-targeting agent that suppresses tubulin polymerization, thereby interfering with mitotic spindle formation and cell division. To verify whether the CA-4-Gefitinib bimolecular prodrug preserves this mechanism upon intracellular activation, we examined the microtubule network architecture in SGC-7901 cells using immunofluorescence staining after treatment with CA-4 alone, the free CA-4/Gefitinib combination, or the prodrug at a single concentration of 7 nM.

**Figure 5:**
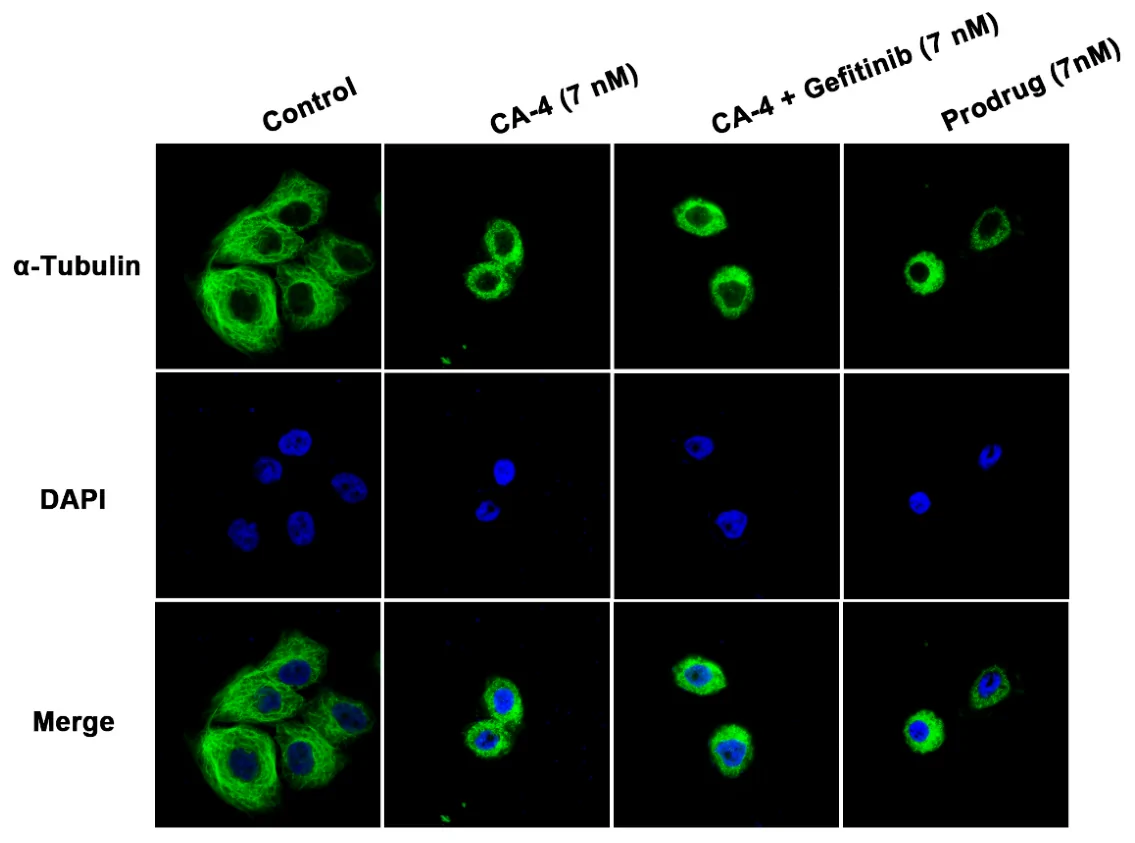
Bimolecular prodrug disrupted the cell microtubule network in SGC-7901 cells. Cell microtubule dynamics were determined by an immunofluorescence staining assay after different treatments either with CA-4, CA-4 + Gefitinib, or bimolecular prodrug in SGC-7901 cells.

As depicted in Figure 5, untreated control cells exhibited an intact and well-organized microtubular cytoskeleton with extended filamentous networks. In contrast, cells exposed to CA-4 alone or the physical mixture of CA-4 and Gefitinib showed marked disruption, characterized by shortened, fragmented, and diffuse tubulin staining, consistent with the expected antimitotic effect. Importantly, treatment with the CA-4-Gefitinib bimolecular prodrug at 7 nM also induced pronounced microtubule disassembly, and the extent of disruption appeared slightly more prominent than that observed with either free CA-4 or the drug combination at equivalent concentrations.

These findings indicate that the prodrug, upon intracellular release of the active CA-4 component, effectively engages its tubulin target and disrupts cytoskeletal integrity. The marginally enhanced effect observed with the prodrug compared to the free agents may reflect improved intracellular delivery or sustained local drug exposure resulting from the covalent prodrug design. Collectively, these data confirm that the bimolecular prodrug retains the core microtubule-destabilizing mechanism of CA-4 while potentially offering a delivery advantage, supporting its utility as a targeted combination therapeutic.

### 3.7. The Bimolecular Prodrug Induces G2/M Phase Cell Cycle Arrest in SGC-7901 Cells

**Figure 6:**
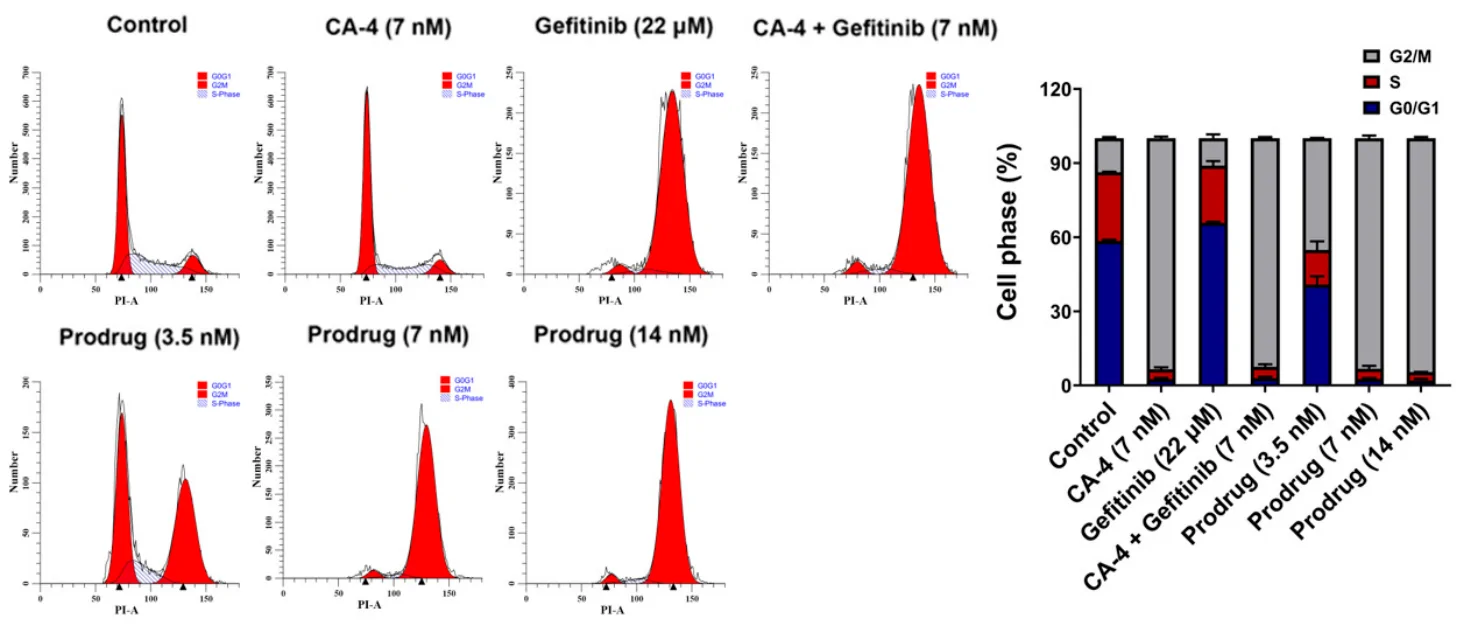
Bimolecular prodrug-induced cell cycle arrest in SGC-7901 cells. Cell cycle was detected by a PI staining assay after different treatments with either Gefitinib, CA-4, CA-4 + Gefitinib, or bimolecular prodrug in SGC-7901 cells.

To further investigate the cellular response to drug treatments, we analyzed the cell cycle distribution in SGC-7901 cells using propidium iodide (PI) staining followed by flow cytometry. As shown in Figure 6, distinct cell cycle arrest patterns were observed depending on the treatment regimen. CA-4 (7 nM) monotherapy predominantly induced G2/M phase arrest, consistent with its mechanism as a microtubule-targeting agent that disrupts mitotic spindle formation. In contrast, Gefitinib (22 μM) treatment resulted in arrest primarily at the G0/G1 phase, aligning with its role as an EGFR signaling inhibitor that suppresses cell cycle progression.

Notably, both the physical combination of CA-4 and Gefitinib (7 nM + 22 μM) and the CA-4-Gefitinib bimolecular prodrug (3.5, 7, and 14 nM) induced cell cycle arrest patterns similar to CA-4 alone, with a predominant accumulation of cells in the G2/M phase. This suggests that the cell cycle-disrupting effect of CA-4 dominates in these combination treatments. Importantly, the bimolecular prodrug induced G2/M phase arrest in a clear dose-dependent manner, with higher prodrug concentrations resulting in greater accumulation of cells in this phase.

These findings provide mechanistic insights into the antiproliferative effects of the prodrug, demonstrating that it effectively delivers biologically active CA-4 intracellularly, leading to cell cycle arrest at the G2/M phase—a key mechanism contributing to its potent anticancer activity.

### 3.8. The Bimolecular Prodrug Promotes Apoptosis in SGC-7901 Cells

**Figure 7:**
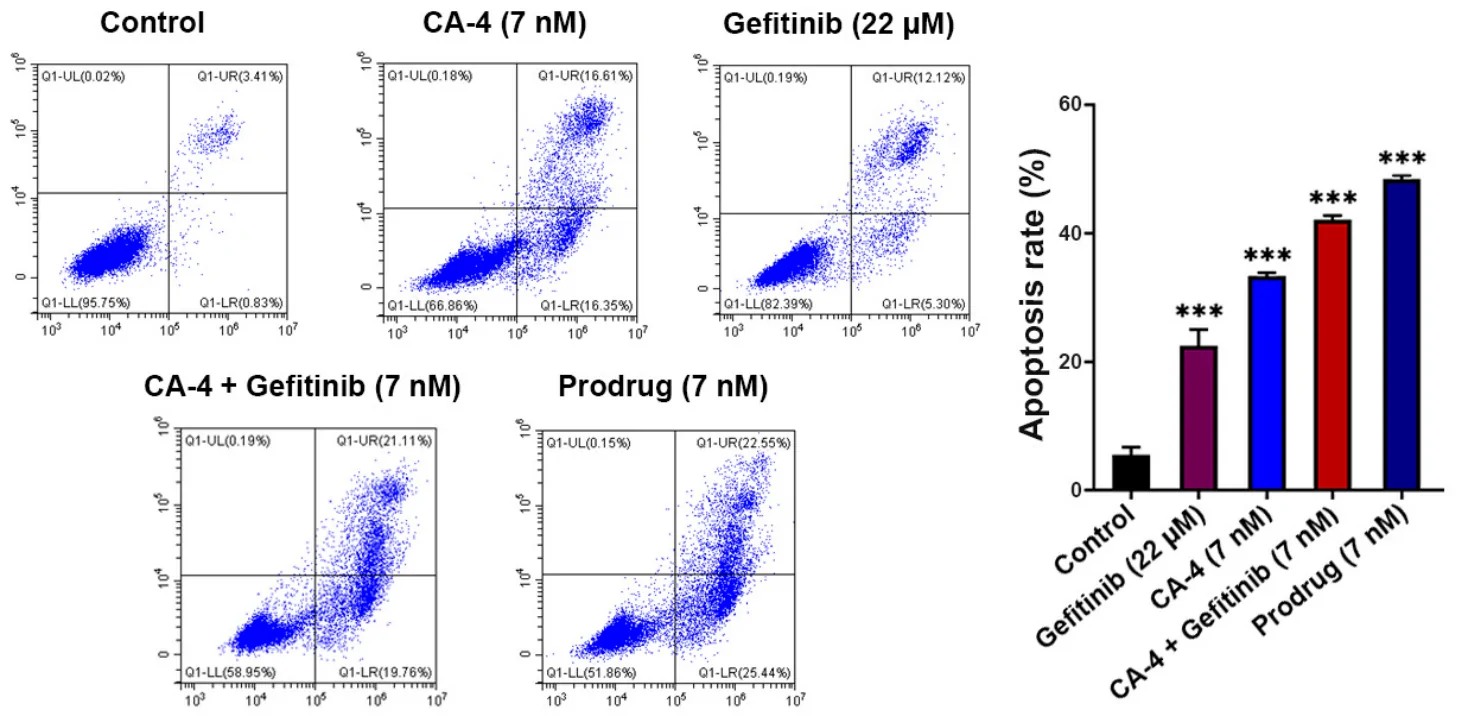
Bimolecular prodrug induced cell apoptosis in SGC-7901 cells. An Annexin V-FITC/PI assay was used to detect cell apoptosis after different treatments with either Gefitinib, CA-4, CA-4 + Gefitinib, or bimolecular prodrug in SGC-7901 cells. Error bars, SEM. *** *p* < 0.001.

To quantitatively assess the cell-death-inducing capacity of the CA-4-Gefitinib bimolecular prodrug, we performed Annexin V-FITC/PI dual staining followed by flow cytometry in SGC-7901 cells. As presented in Figure 7, untreated control cells exhibited a basal level of apoptosis, whereas exposure to CA-4 (7 nM) or Gefitinib (22 μM) alone resulted in a moderate but significant increase in the percentage of apoptotic cells, consistent with their individual cytotoxic mechanisms.

The physical combination of CA-4 and Gefitinib produced a markedly higher apoptotic rate than either monotherapy, indicative of a cooperative pro-apoptotic interaction between the two agents. Notably, treatment with the CA-4-Gefitinib bimolecular prodrug at an equivalent concentration of 7 nM further elevated the apoptotic population, surpassing the level achieved by the free drug combination. This enhanced effect suggests that the covalently linked prodrug design facilitates synchronized intracellular delivery and release of both active components, thereby promoting more efficient engagement of apoptotic signaling cascades compared to co-administration of the unconjugated agents.

Collectively, these findings demonstrate that the bimolecular prodrug not only retains but also amplifies the pro-apoptotic efficacy of the CA-4/Gefitinib combination. The superior apoptosis-inducing activity observed with the prodrug reinforces its potential as a targeted therapeutic strategy capable of driving robust programmed cell death in gastric cancer cells, while also supporting the mechanistic rationale for its enhanced antitumor performance observed in subsequent assays.

### 3.9. The Bimolecular Prodrug Suppresses SGC-7901 Cell Migration

**Figure 8:**
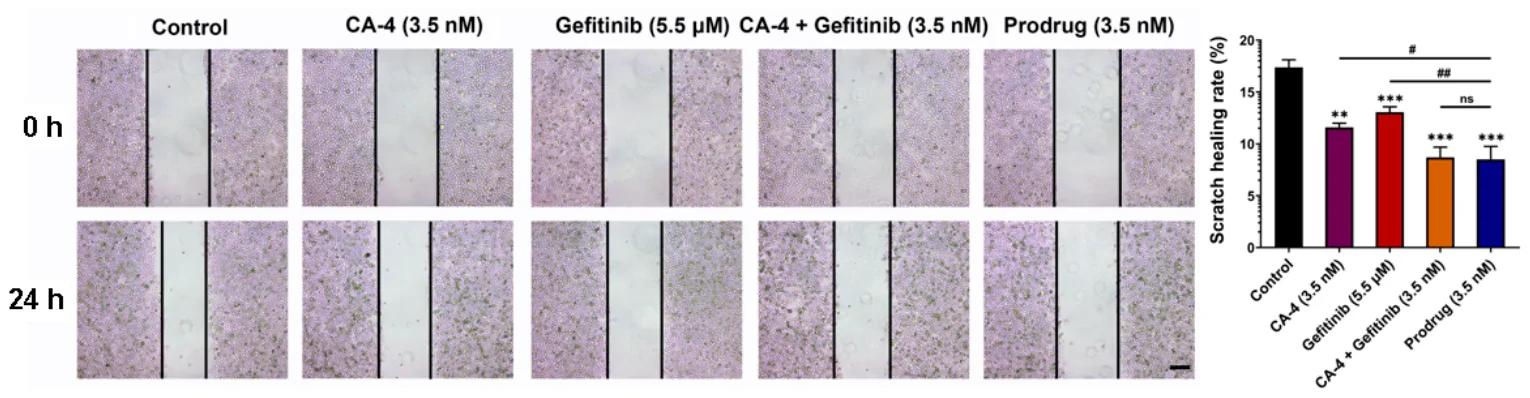
Bimolecular prodrug inhibited cell migration in SGC-7901 cells. Cell migration was determined through a wound-healing assay after different treatments either with Gefitinib, CA-4, CA-4 + Gefitinib, or bimolecular prodrug in SGC-7901 cells. Error bars, SEM. ns, not significant; ** *p* < 0.01; *** *p* < 0.001; # *p* < 0.05; ## *p* < 0.01.

Tumor cell dissemination remains a principal determinant of cancer-related mortality, underscoring the critical need for therapeutic interventions that effectively impede cell migration. To investigate whether the CA-4-Gefitinib bimolecular prodrug exerts inhibitory effects on the motile behavior of gastric cancer cells, we conducted a wound-healing assay in SGC-7901 monolayers. As illustrated in Figure 8, treatment with CA-4 (3.5 nM) or Gefitinib (5.5 μM) alone significantly retarded wound closure relative to the untreated control, reflecting the individual anti-migratory activities of both agents. Co-administration of the two free drugs at equivalent concentrations produced a more pronounced suppression of cell movement, suggesting a cooperative effect on migration-related pathways.

Remarkably, the CA-4-Gefitinib bimolecular prodrug at 3.5 nM exhibited the most robust inhibition of wound healing among all tested groups, outperforming both monotherapies and the physical drug combination. This superior anti-migratory efficacy is likely attributable to the concerted intracellular action of CA-4—which disrupts microtubule dynamics essential for cytoskeletal remodeling and cell motility—and Gefitinib, which blocks EGFR-driven signaling cascades that promote directional migration. The covalent prodrug design ensures that both pharmacological activities are delivered concomitantly to the same target cells, thereby maximizing the cooperative suppression of migratory potential.

Collectively, these findings indicate that the bimolecular prodrug not only preserves but also potentiates the anti-migratory effects of the individual agents. The enhanced activity observed at a relatively low concentration (3.5 nM) highlights the therapeutic promise of this prodrug strategy in counteracting tumor invasion and metastasis, in addition to its direct cytotoxic actions.

### 3.10. The Bimolecular Prodrug Inhibits Tube Formation in HUVECs

**Figure 9:**
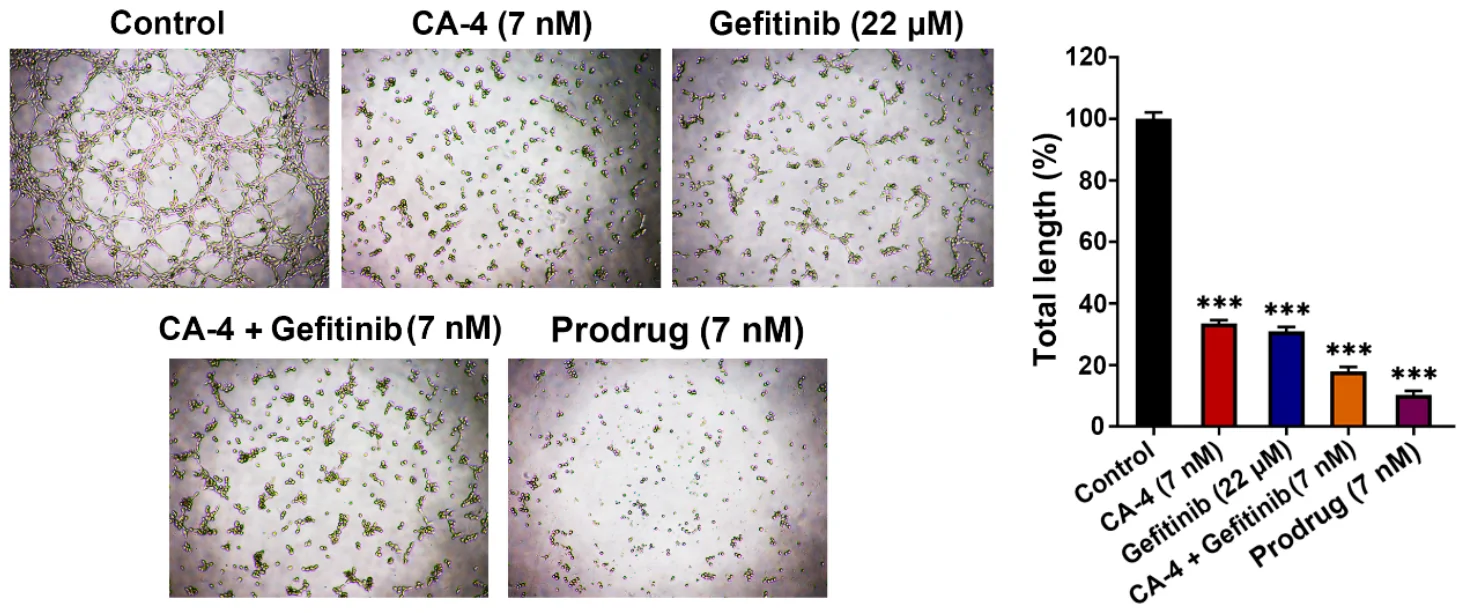
Bimolecular prodrug inhibited tube formation in HUVECs. Angiogenesis was detected by a tube formation assay after different treatments either with Gefitinib, CA-4, CA-4 + Gefitinib, or bimolecular prodrug in HUVECs; *** *p* < 0.001.

Angiogenesis constitutes a hallmark of tumor progression, providing essential nutritional and metastatic routes for malignant growth. To evaluate the anti-angiogenic potential of the CA-4-Gefitinib bimolecular prodrug, we performed a tube formation assay using human umbilical vein endothelial cells (HUVECs) cultured on Matrigel. As depicted in Figure 9, untreated control cells formed an extensive and well-organized tubular network, indicative of robust angiogenic capacity. Treatment with CA-4 (7 nM) or Gefitinib (22 μM) alone significantly reduced tube formation, consistent with the known anti-vascular activities of both agents.

Co-administration of free CA-4 and Gefitinib resulted in a more pronounced disruption of the tubular architecture, suggesting an additive or synergistic inhibitory effect on endothelial cell organization. Strikingly, the CA-4-Gefitinib bimolecular prodrug at 7 nM elicited the most substantial suppression of tube formation among all treatment groups, with the remaining tubular structures appearing sparse, fragmented, and largely disconnected. This superior anti-angiogenic efficacy indicates that the covalently linked prodrug design facilitates coordinated delivery of both active pharmacophores to endothelial cells, enabling more effective interference with the multiple signaling pathways governing vascular morphogenesis.

These findings demonstrate that the bimolecular prodrug not only retains but also amplifies the anti-angiogenic activities of its constituent agents. Given the critical dependence of solid tumors on neovascularization, the potent tube-formation inhibitory effect observed with the prodrug further supports its therapeutic potential as a multifaceted anticancer strategy, combining direct cytotoxicity with robust disruption of the tumor vasculature.

### 3.11. Pharmacokinetics Study of the Bimolecular Prodrug

To investigate the pharmacokinetic properties of the CA-4-Gefitinib bimolecular prodrug and whether CA-4 and Gefitinib analog can be released from the CA-4-Gefitinib bimolecular prodrug in vivo, we administered the prodrug to SD rats via tail vein injection at a dose of 11.5 mg/kg (3.7 mg/kg of CA-4 and 5.3 mg/kg of Gefitinib). Blood samples were collected at specific time points for UPLC-MS analysis to detect the presence of the CA-4-Gefitinib bimolecular prodrug, CA-4, and Gefitinib analog in plasma. Animals treated with 3.7 mg/kg of CA-4 and 5.3 mg/kg of Gefitinib were used as a comparison for pharmacokinetic analysis. The findings revealed that CA-4 was detected in CA-4-treated animals, Gefitinib was detected in Gefitinib-treated animals, and CA-4, Gefitinib analog, and CA-4-Gefitinib bimolecular prodrug were detected in blood samples of animals administered the CA-4-Gefitinib bimolecular prodrug. Table 2 shows the corresponding pharmacokinetic parameters calculated through non-compartmental analysis (mean ± SD). Figure S3 depicts the mean plasma concentrations of CA-4 and Gefitinib (analog) after intravenous administration of CA-4-Gefitinib bimolecular prodrug, CA-4, and Gefitinib, respectively. The pharmacokinetic analysis of CA-4 in the CA-4-Gefitinib bimolecular prodrug revealed an AUC_0–∞_ value of 1208 ± 101 h·ng/mL, a CL value of 3119 ± 266 mL/h/kg, and a T_1/2_ value of 0.83 ± 0.35 h. The C_max_ value for CA-4 in the CA-4-Gefitinib bimolecular prodrug (5673 ± 330 ng/mL) was significantly higher than that observed with the CA-4 solution (1571 ± 233 ng/mL). Furthermore, the T_1/2_ of CA-4 in the CA-4-Gefitinib bimolecular prodrug (0.83 ± 0.35 h) exhibited a notable increase compared to that of the CA-4 solution (0.67 ± 0.25 h), indicating a slower clearance from plasma despite lower initial concentrations. Additionally, the CL for CA-4 in the CA-4-Gefitinib bimolecular prodrug (3119 ± 266 mL/h/kg) was also significantly lower than that for the CA-4 solution (7681 ± 1369 mL/h/kg). Notably, there was a substantial increase in AUC_0–∞_ for CA-4 in the CA-4-Gefitinib bimolecular prodrug (1208 ± 101 h·ng/mL) compared to that of the CA-4 solution (49,904 ± 916 h·ng/mL). These results indicated that the CA-4-Gefitinib bimolecular prodrug could significantly reduce the exposure concentration and prolong the circulation time of CA-4 in blood compared with the pharmacokinetic behavior of total CA-4 in plasma after injection of CA-4 solution in rats, which could be conducive to prolonging the interaction time for the CA-4-Gefitinib bimolecular prodrug releasing CA-4 in tumor cells, leading to better antitumour efficacy and lower toxicity. Additionally, when comparing the pharmacokinetic behavior of total Gefitinib in plasma following the injection of Gefitinib solution in rats, there was no significant improvement in the AUC_0–∞_, CL, and T_1/2_ of the total Gefitinib analog in the CA-4-Gefitinib bimolecular prodrug.

### 3.12. In Vivo Antitumour Efficacy of the Bimolecular Prodrug

**Figure 10:**
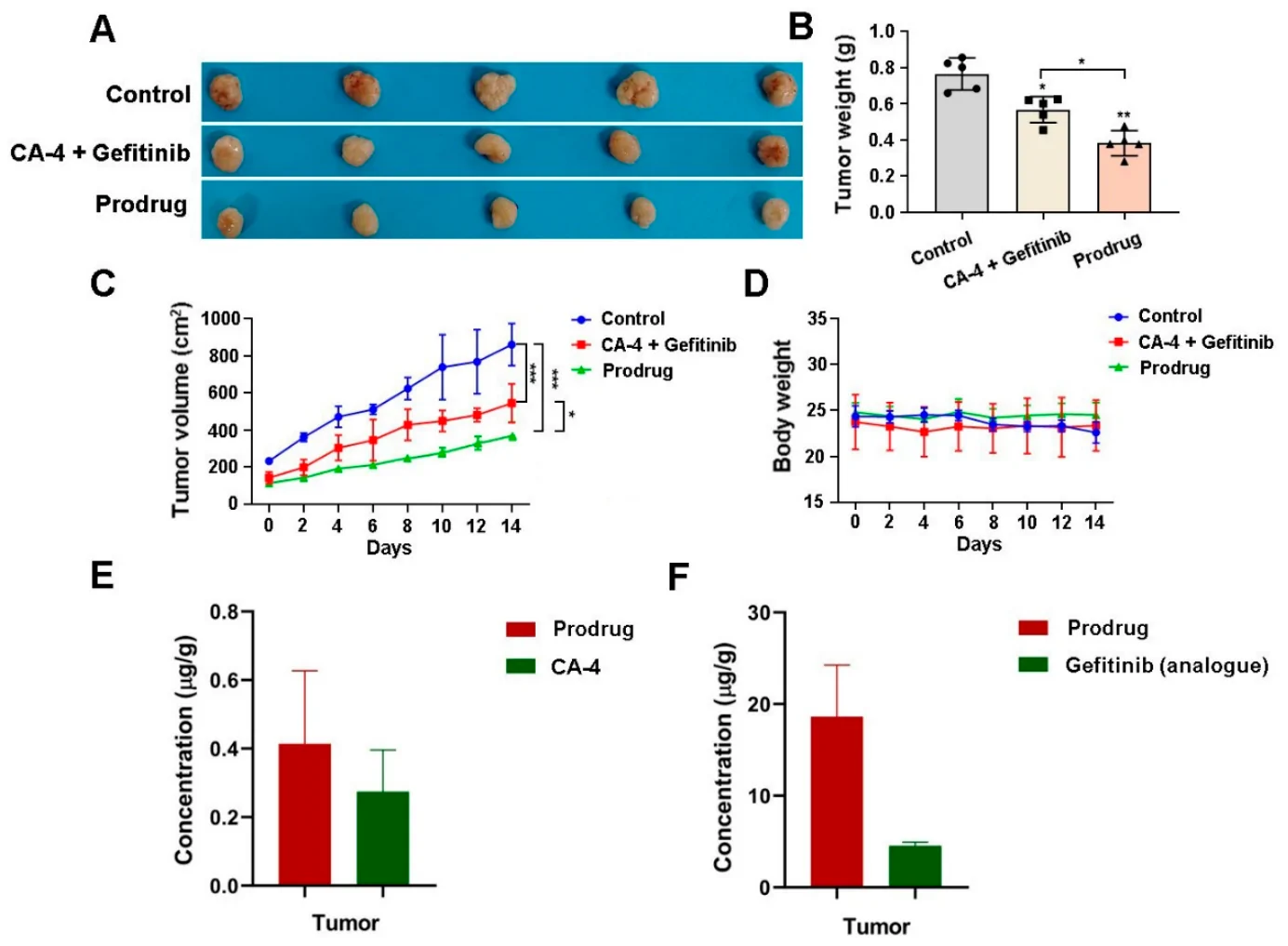
Antitumor activity of bimolecular prodrug in an SGC-7901 xenograft mouse model. (**A**) Image of the tumors 2 weeks after the initial administration. (**B**) Weight of tumors after treatment for 2 weeks. (**C**) Changes in tumor volume in mice during the administration. (**D**) Changes in mouse body weight during the administration. (**E**) The concentration of CA-4 in tumor tissue. (**F**) The concentration of Gefitinib (analog) in tumor tissue.

The in vivo antitumor activity of the CA-4-Gefitinib bimolecular prodrug was systematically evaluated in a murine xenograft model. As depicted in Figure 10A–C, administration of the prodrug at a dose of 46.0 mg/kg resulted in significant tumor growth suppression compared to the vehicle control. Importantly, the antitumor efficacy of the bimolecular prodrug surpassed that achieved by the positive control—a combination of free CA-4 (15.0 mg/kg) and Gefitinib (21.2 mg/kg)—suggesting a therapeutic advantage of the prodrug platform over the conventional co-administration of the two drugs.

In addition to its enhanced efficacy, the safety profile of the prodrug was evaluated by monitoring changes in mouse body weight throughout the study. As shown in Figure 10D, no significant body weight loss was observed in mice treated with the bimolecular prodrug, indicating favorable tolerability and minimal systemic toxicity associated with the formulation.

To elucidate the mechanistic basis for the superior antitumor performance of the prodrug, tumor tissues were excised from both the positive control and prodrug-treated groups after two weeks of treatment. Subsequent quantitative analysis via UPLC-MS revealed significantly higher intratumoral concentrations of the active moieties in the prodrug group: CA-4 reached 0.45 μg/g, while the Gefitinib analog was detected at 20 ng/g. In contrast, tumor tissues from the combination therapy group contained only 0.25 μg/g of CA-4 and 5 ng/g of Gefitinib (Figure 10E).

The markedly elevated tumor deposition of both active agents suggests that the bimolecular prodrug facilitates enhanced tumor targeting and/or retention compared to the free drug combination. This improved biodistribution profile likely contributes to the observed enhancement in antitumor activity, supporting the prodrug strategy as an effective means to optimize drug delivery and therapeutic outcomes. Further studies are warranted to fully decipher the in vivo release kinetics and tumor-specific activation mechanisms of the prodrug.

### 3.13. In Vivo Safety Evaluation

To comprehensively assess the biosafety profile of the CA-4-Gefitinib bimolecular prodrug, a systematic in vivo toxicity study was conducted in healthy ICR mice. The animals (*n* = 3) received intravenous injections of the prodrug at a therapeutic dose of 46.0 mg/kg every other day. After a 10-day treatment period, major organs and blood samples were collected for detailed histopathological and hematological analyses.

**Figure 11:**
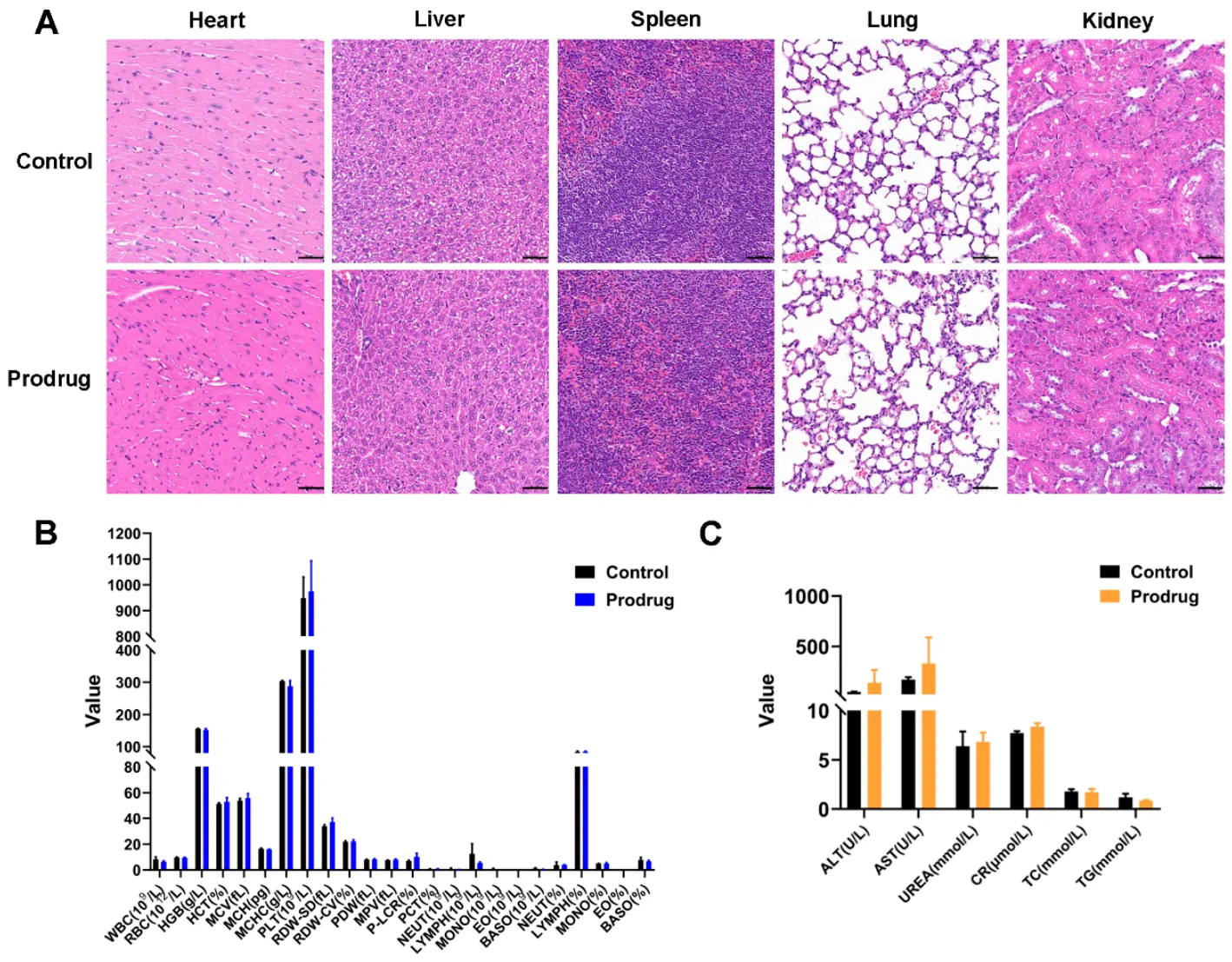
The safety of bimolecular prodrug in vivo. (**A**) Representative images of H&E-stained vital organs, including liver, lung, heart, kidney, and spleen. Scale bar = 50 μm. (**B**) Biosafety of bimolecular prodrug assessed by hematology tests. (**C**) Biosafety of bimolecular prodrug assessed by blood biochemistry tests. (mean ± SD, *n* = 3).

Histological examination *via* hematoxylin and eosin (H&E) staining revealed no evident signs of tissue damage or pathological alterations in major organs—including the heart, liver, spleen, lungs, and kidneys—from the prodrug-treated group compared to the control group (Figure 11A). This suggests that the prodrug does not induce acute organ toxicity at the administered dosage.

Further supporting its safety profile, complete blood analysis showed no statistically significant differences in key hematological parameters between the prodrug-administered mice and the control animals (Figure 11B). In addition, serum biochemistry tests were performed to evaluate potential hepatorenal toxicity. As illustrated in Figure 11C, the levels of liver function markers (alanine aminotransferase, ALT; aspartate aminotransferase, AST) and kidney function indicators (creatinine, CRE; blood urea nitrogen, BUN) all remained within normal physiological ranges and were comparable to those of the control group.

Collectively, the results from histopathology, hematology, and serum biochemistry analyses provide compelling evidence for the favorable in vivo safety and biocompatibility of the CA-4-Gefitinib bimolecular prodrug, underscoring its potential for further therapeutic development.

## 4. Conclusions

Combination chemotherapy, which engages complementary oncogenic pathways, offers a compelling strategy for achieving synergistic antitumor effects. However, the clinical potential of promising drug pairs—such as the microtubule-targeting agent CA-4 and the EGFR inhibitor Gefitinib—is frequently constrained by disparate pharmacokinetic profiles, suboptimal tumor accumulation, and asynchronous delivery to malignant tissues. To surmount these obstacles, we rationally designed and synthesized a redox-responsive bimolecular prodrug that covalently conjugates CA-4 and Gefitinib via a disulfide-based linker. The structural integrity of the conjugate was unequivocally confirmed through comprehensive spectroscopic characterization.

The prodrug demonstrated high stability under physiological conditions while undergoing rapid and selective cleavage in the presence of elevated glutathione levels, thereby enabling synchronized release of both active agents within cancer cells. This tumor-microenvironment-activated delivery mechanism translated into broad-spectrum cytotoxicity across multiple cancer cell lines, with markedly enhanced selectivity for malignant over normal cells compared to free drug regimens. We note that CA-4 exhibits inherently stronger nanomolar antiproliferative potency than Gefitinib (micromolar range) in SGC-7901 gastric adenocarcinoma cells; thus, CA-4 represents the primary mediator of direct cytotoxicity. Nevertheless, the covalently tethered Gefitinib moiety confers additional biological functions, including suppression of EGFR-mediated tumor cell migration and angiogenesis, which cannot be achieved by CA-4 monotherapy alone. In SGC-7901 gastric adenocarcinoma models, the prodrug at a single equivalent concentration consistently outperformed both monotherapies and the physical drug combination across a range of functional endpoints, including inhibition of clonogenic survival, disruption of microtubule integrity, induction of apoptosis, suppression of cell migration, and blockade of angiogenic tube formation.

Pharmacokinetic profiling further revealed that the prodrug formulation significantly prolongs systemic circulation and reduces premature drug exposure, thereby enhancing the likelihood of tumor-selective accumulation and bioactivation. In a murine xenograft model, the prodrug achieved superior antitumor efficacy relative to the free drug combination, accompanied by a favorable safety profile as evidenced by histopathological, hematological, and serum biochemical analyses. It should be acknowledged that this study lacks a key control prodrug containing only CA-4 (without the Gefitinib-like fragment), which would help to further distinguish the contribution of the Gefitinib pharmacophore and clarify whether improved cell permeability arises solely from the linker structure. Synthesis and systematic biological evaluation of this CA-4 mono-prodrug will be prioritized in our follow-up investigations.

Collectively, this “two-in-one” prodrug strategy—anchored in a tumor-redox-responsive drug–drug conjugate—provides a robust and generalizable platform for maximizing synergistic efficacy while minimizing off-target toxicity. Our findings establish the CA-4-Gefitinib bimolecular prodrug as a promising therapeutic candidate for gastric cancer and offer a rational blueprint for the future development of targeted combination chemotherapies.

## Figures and Tables

**Table 1:** In vitro anticancer activity (IC_50_ in nM) of the compounds.

Cell Lines	Gefitinib (μM)	CA-4 (nM)	Gefitinib + CA-4 (nM)	CA-4-Gefitinib Prodrug (nM)
SGC-7901	22.15 ± 2.16	13.00 ± 1.19	11.34 ± 0.74	7.17 ± 0.65
HCT-116	33.16 ± 1.32	26.69 ± 2.24	26.02 ± 1.42	13.82 ± 0.86
HepG2	13.37 ± 0.73	17.72 ± 0.15	16.24 ± 0.21	14.23 ± 0.05
MCF-7	32.06 ± 2.83	18.36 ± 1.96	17.57 ± 0.77	11.91 ± 0.16
A549	9.96 ± 0.53	44.11 ± 2.00	36.67 ± 1.56	30.89 ± 0.93
HCC827	0.46 ± 0.05	11.95 ± 1.72	8.19 ± 0.75	15.04 ± 0.72
NCI-H1975	14.75 ± 1.45	7.90 ± 1.71	8.11 ± 0.69	7.92 ± 0.48
HUVEC	25.25 ± 5.41	204.30 ± 55.86	140.00 ± 39.35	701.20 ± 115.62
Selectivity (HUVEC/SGC-7901)	1	15	12	98

**Table 2:** Plasma pharmacokinetic parameters after intravenous administration of CA-4, Gefitinib or bimolecular prodrug in rats.

Parameters	CA-4		Gefitinib (Analog)	
	CA-4	Prodrug	Gefitinib	Prodrug
T_1/2_(h)	0.67 ± 0.25	0.83 ± 0.35	19.28 ± 2.85	10.16 ± 0.22
Cmax(ng/mL)	1571 ± 233	5673 ± 330	497.39 ± 66.6	67.93 ± 7.61
AUC_0–t_(hng/mL)	489.5 ± 86.3	1200 ± 101	654.9 ± 107.6	130.83 ± 15.2
AUC_0–∞_(h ng/mL)	499.04 ± 91.6	1208 ± 101	898.2 ± 166.7	154.24 ± 18.7
Vz (mL/kg)	7161 ± 1871	3665 ± 1449	165,009 ± 12,221	508,791 ± 63,129
CL (mL/h/kg)	7681 ± 1369	3119 ± 266	6026 ± 1013	34,680 ± 3957

## Data Availability

The raw data supporting the conclusions of this article will be made available by the authors on request.

## Supplementary Materials

The following supporting information can be downloaded at: https://www.mdpi.com/article/10.3390/antiox15091214/s1. Figure S1. The interaction analysis of CA-4 and gefitinib in SGC-7901 cells. The cell viability was detected by a CCK-8 assay 48 h after cells were treated with CA-4 and gefitinib, then the online SynergyFinder software was used to estimate the drug interaction by ZIP synergy scores. Figure S2. Representative chromatograms and ESI/MS spectrums of CA-4-Gefitinib bimolecular prodrug incubated with GSH for 0 h and 2 h. Figure S3. (A) Mean plasma concentration-time curves of CA-4 after intravenous administration of CA-4-Gefitinib bimolecular prodrug and CA-4 to rats; (B) Mean plasma concentration-time curves of Gefitinib (analogue) after intravenous administration of CA-4-Gefitinib bimolecular prodrug and Gefitinib to rats. Figure S4. HRMS, ^1^H-NMR, and ^13^C-NMRspectra of CA-4-Gefitinib bimolecular prodrug. Figure S5. Magnified ^13^C NMR spectrum. Figure S6. Magnified ^13^C-NMR spectrum extracted from Figure S5.

## Abbreviations

The following abbreviations are used in this manuscript:

CA-4	Combretastatin A-4
EGFR	Epidermal growth factor receptor
GSH	Glutathione
ERK	Extracellular signal-regulated kinase
PARP	Poly (ADP-ribose) polymerase
Bcl-2	B-cell lymphoma 2
MAPK	Mitogen-activated protein kinase
PI3K	Phosphoinositide 3-kinase
AKT	Protein kinase B
VDA	Vascular disrupting agent
NSCLC	Non-small cell lung cancer
G2/M	Gap 2/Mitosis phase
IC_50_	Half-maximal inhibitory concentration
SI	Safety index
NMR	Nuclear magnetic resonance
DMAP	4-Dimethylaminopyridine
DIPEA	*N*,*N*-Diisopropylethylamine
TFA	Trifluoroacetic acid
THF	Tetrahydrofuran
DCM	Dichloromethane
DMF	*N*,*N*-Dimethylformamide
PBS	Phosphate-buffered saline
HUVEC	Human umbilical vein endothelial cell
SGC-7901	Human gastric adenocarcinoma cell line
MCF-7	Human breast cancer cell line
HepG2	Human hepatocellular carcinoma cell line
HCT-116	Human colorectal carcinoma cell line
A549	Human non-small cell lung cancer cell line
HCC827	Human lung adenocarcinoma cell line (EGFR exon 19 deletion)
NCI-H1975	Human lung adenocarcinoma cell line (EGFR T790M/L858R)
AUC	Area under the concentration-time curve
CL	Clearance
Cmax	Maximum plasma concentration
T_1_/_2_	Half-life
H&E	Hematoxylin and eosin
ALT	Alanine aminotransferase
AST	Aspartate aminotransferase
CRE	Creatinine
BUN	Blood urea nitrogen
SD rats	Sprague-Dawley rats
ICR mice	Institute of Cancer Research mice
NSG mice	NOD-SCID IL2Rγ-null mice
