# Supplementary material for: Engineering a Redox-Responsive Bimolecular Prodrug for Targeted Gastric Cancer Combination Therapy: Design, Synthesis, and In Vivo Evaluation
Source: Antioxidants (Basel). 2026 Sep 21;15(9):1214. doi: 10.3390/antiox15091214 (PMC13603236; doi:10.3390/antiox15091214)
Supplement: Supplementary file 1 [file antioxidants-15-01214-s001.zip › antioxidants-4518474-supplementary.pdf]

Supplementary data

Engineering a redox-responsive bimolecular prodrug for targeted gastric cancer combination therapy: Design, synthesis, and in vivo evaluation

Chao Wang [1,2], Yutao Xiu [1,2], Yujing Zhang [1,2], Jiazhen Xu [1,2], Yanhong Wang [3,*] and Dongming Xing [1,2,4,*]

1 Cancer Institute, The Affiliated Hospital of Qingdao University, Qingdao University, Qingdao 266071, China; wangchao@qdu.edu.cn (C.W.); xiuyutao@qdu.edu.cn (Y.X.); yujingzhang666@163.com (Y.Z.); xujiazhen1@qdu.edu.cn (J.X.)
2 Qingdao Cancer Institute, Qingdao University, Qingdao 266071, China
3 School of Nursing, Qingdao Binhai University, Qingdao 266555, China
4 School of Life Sciences, Tsinghua University, Beijing 100084, China
* Correspondence: wangyh@qdu.edu.cn (Y.W.); xingdongming@qdu.edu.cn (D.X.)

The interaction of CA-4 and Gefitinib in SGC-7901 cells

To improve drug efficacy and reduce the side effects of chemotherapy in the clinic, we tested whether CA-4 could synergize with Gefitinib in the treatment of gastric cancer. According to the IC_{50} values of CA-4 and Gefitinib, we designed a dose-range experiment and detected cell viability by CCK-8 assay, in which dose-escalating CA-4 (0 - 13 nM) and Gefitinib (0 - 22 μM) were added to SGC-7901 cells alone or in combination. The online SynergyFinder software was then used to calculate the ZIP synergy scores based on the viability index (**Fig. S1**). The results showed that the average drug interaction score was 5.089 in SGC-7901 cells. Collectively, the combination of CA-4 and Gefitinib displayed significantly additive synergistic effects in SGC-7901 cells (ZIP synergy scores < -10 were considered to be antagonistic and scores ≥ -10 were considered to be additive synergistic.).

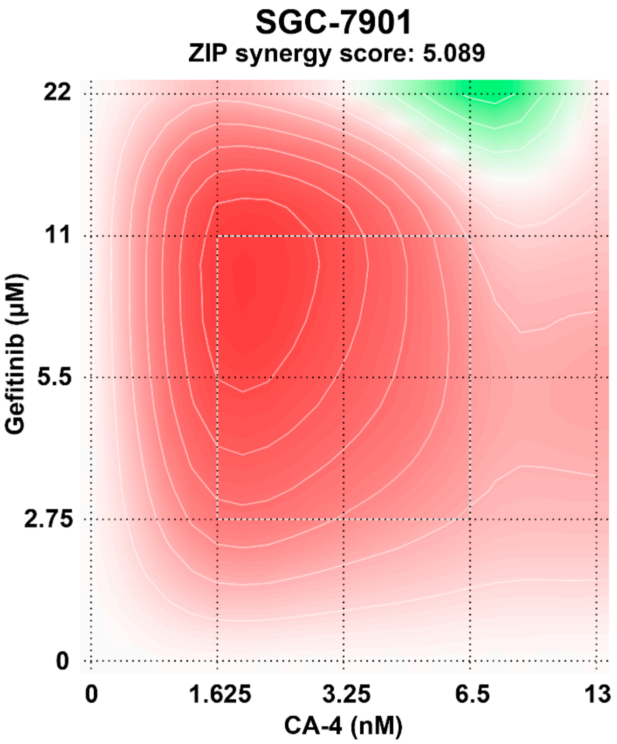

Figure S1. The interaction analysis of CA-4 and gefitinib in SGC-7901 cells. Cell viability was detected by a CCK-8 assay 48 h after cells were treated with CA-4 and gefitinib, then the online SynergyFinder software was used to estimate the drug interaction by ZIP synergy scores.

The anticipated release of CA-4 and Gefitinib was confirmed by reverse-phase UPLC-MS analyses (**Fig.S2**). The intact CA-4-Gefitinib bimolecular prodrug was eluted at 28.5 min in the UPLC chromatogram (**Fig. S2A**) and the corresponding peak in the HRMS spectrum (**Fig. S2B**) was at 968.2760 m/z $[M + H]^+$. When GSH was added to the CA-4-Gefitinib bimolecular prodrug for 2 h, the CA-4-Gefitinib bimolecular prodrug was cleaved by GSH. Due to the chemical reaction involved, this cleavage underwent intramolecular cyclization, followed by ring-forming condensation, resulting in the liberation of CA-4 and Gefitinib. The peak at 28.5 min, corresponding to the CA-4-Gefitinib bimolecular prodrug, decreased dramatically, and new strong fragment peaks appeared that eluted at 6.35 min and 28.6 min, well matched with free CA-4 and Gefitinib, respectively, corresponding to the peak intensities at 317.1374 m/z (**Fig. S2C**) and 446.17328 m/z $[M + H]^+$ (**Fig. S2D**), respectively, in HRMS spectral analysis. Together, the disulfide bond present in the CA-4-Gefitinib bimolecular prodrug was demonstrated to be cleaved by GSH.

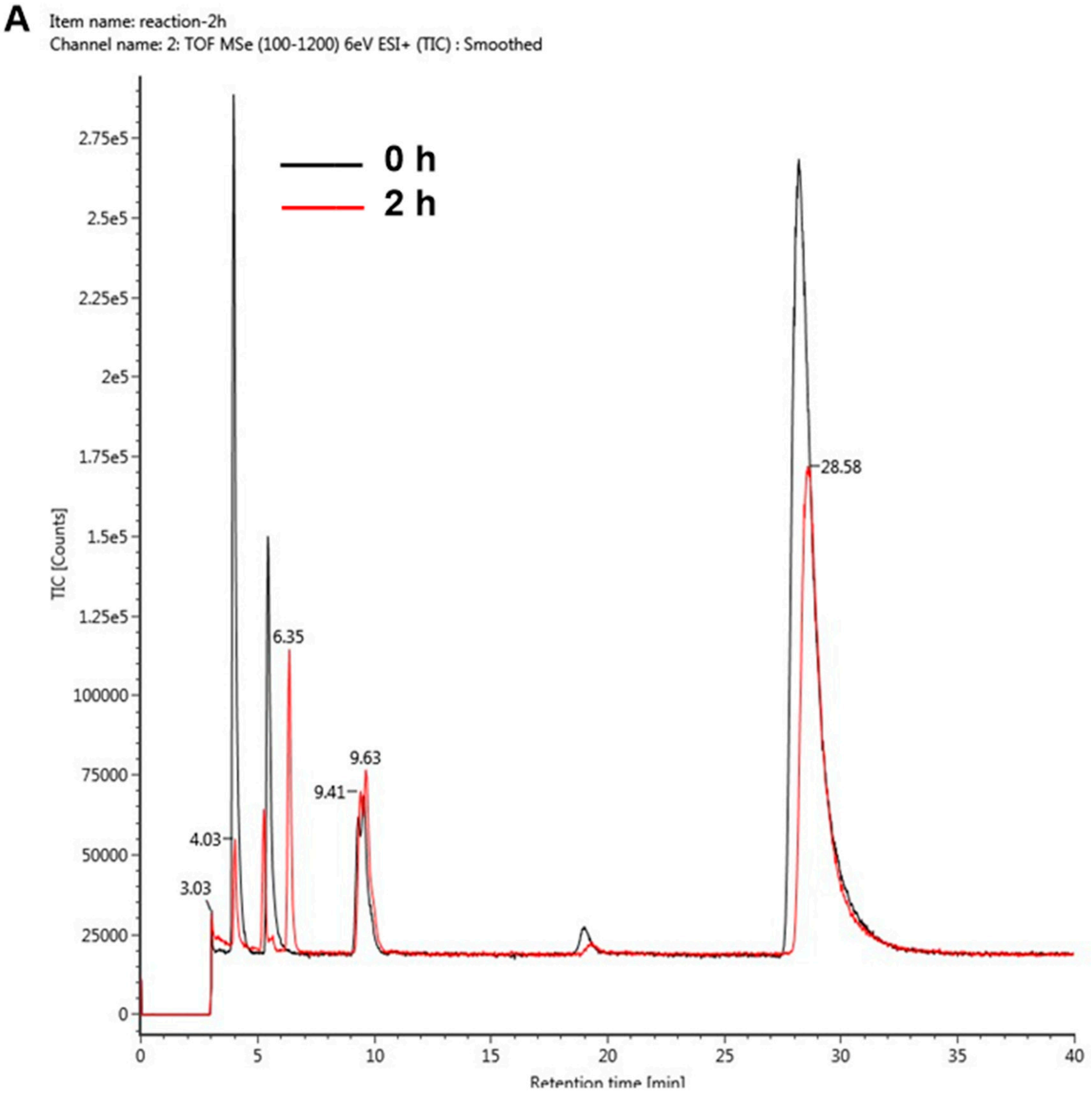

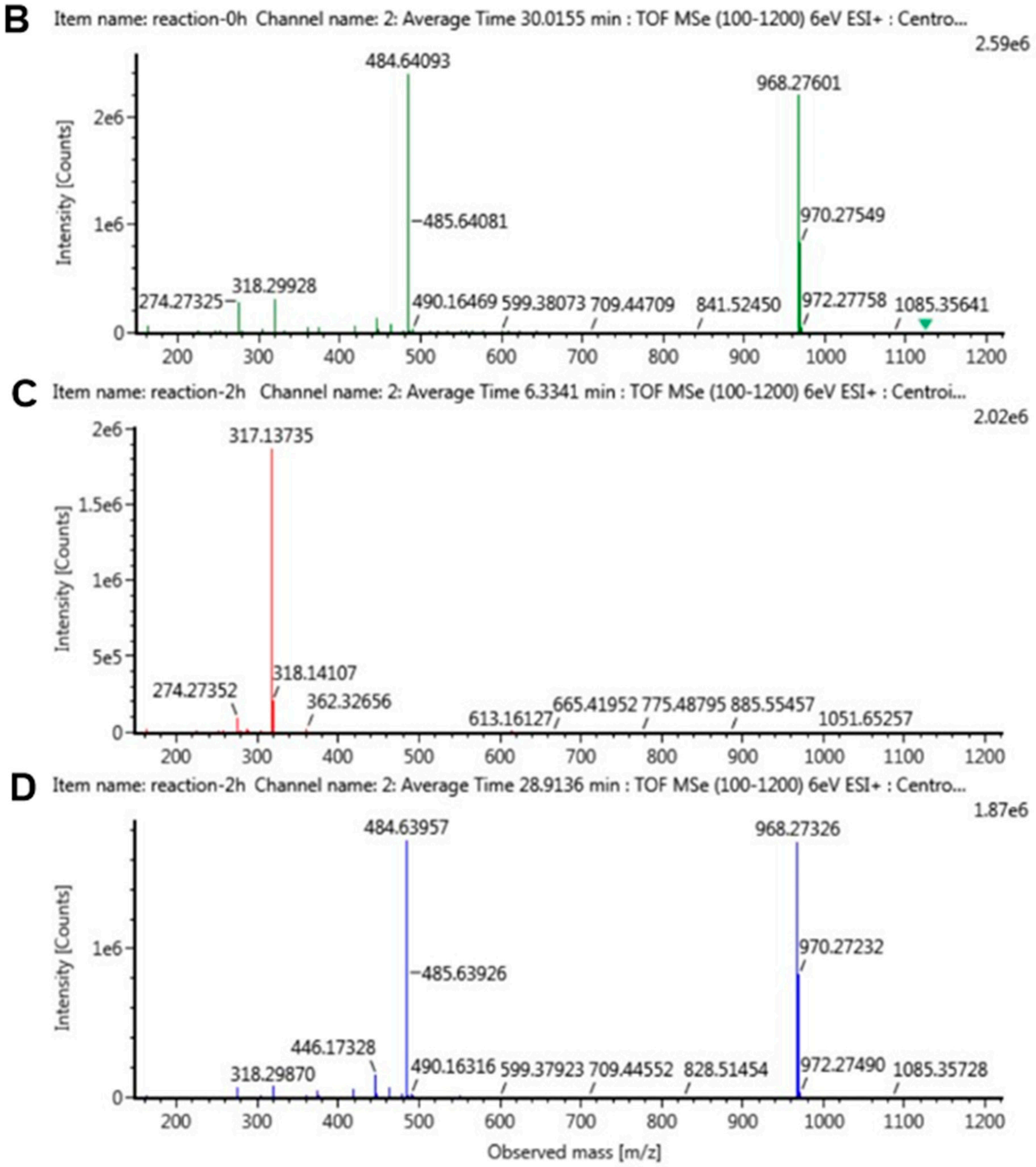

Figure S2. Representative chromatograms and ESI/MS spectra of CA-4-Gefitinib bimolecular prodrug incubated with GSH for 0 h and 2 h.

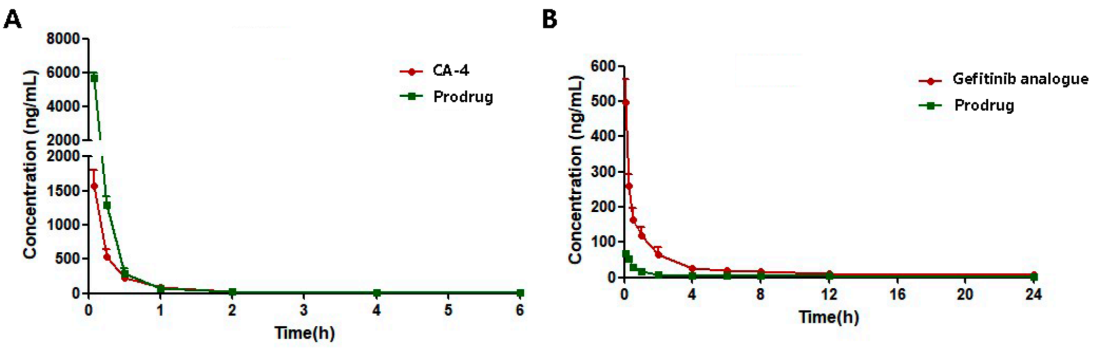

Figure S3. (A) Mean plasma concentration-time curves of CA-4 after intravenous administration of CA-4-Gefitinib bimolecular prodrug and CA-4 to rats; (B) Mean plasma concentration-time curves of Gefitinib (analogue) after intravenous administration of CA-4-Gefitinib bimolecular prodrug and Gefitinib to rats.

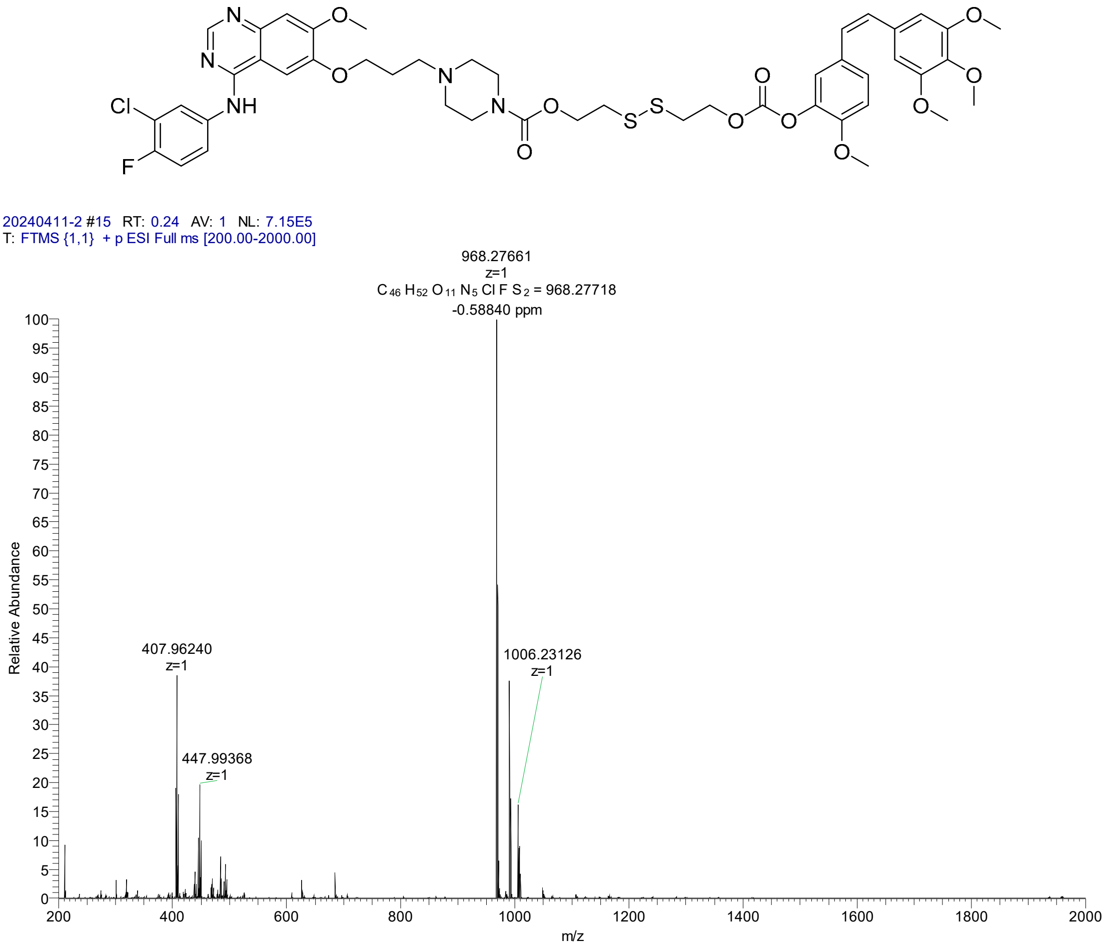

Figure S4. HRMS, ^{1}H-NMR, and ^{13}C-NMR spectra of CA-4-Gefitinib bimolecular prodrug

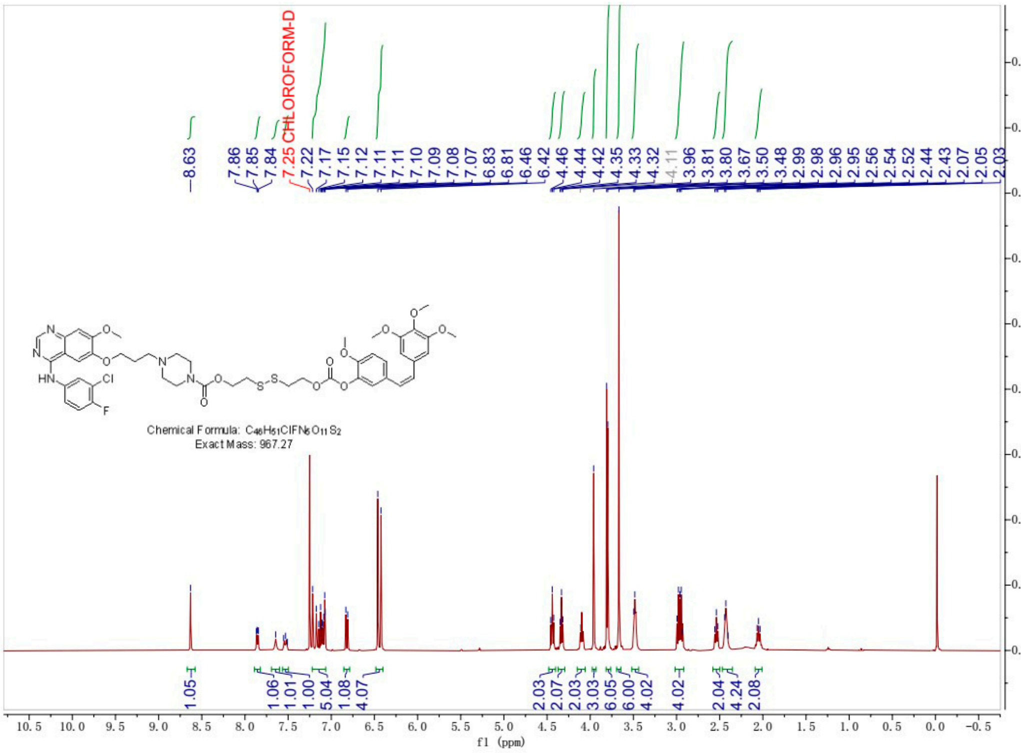

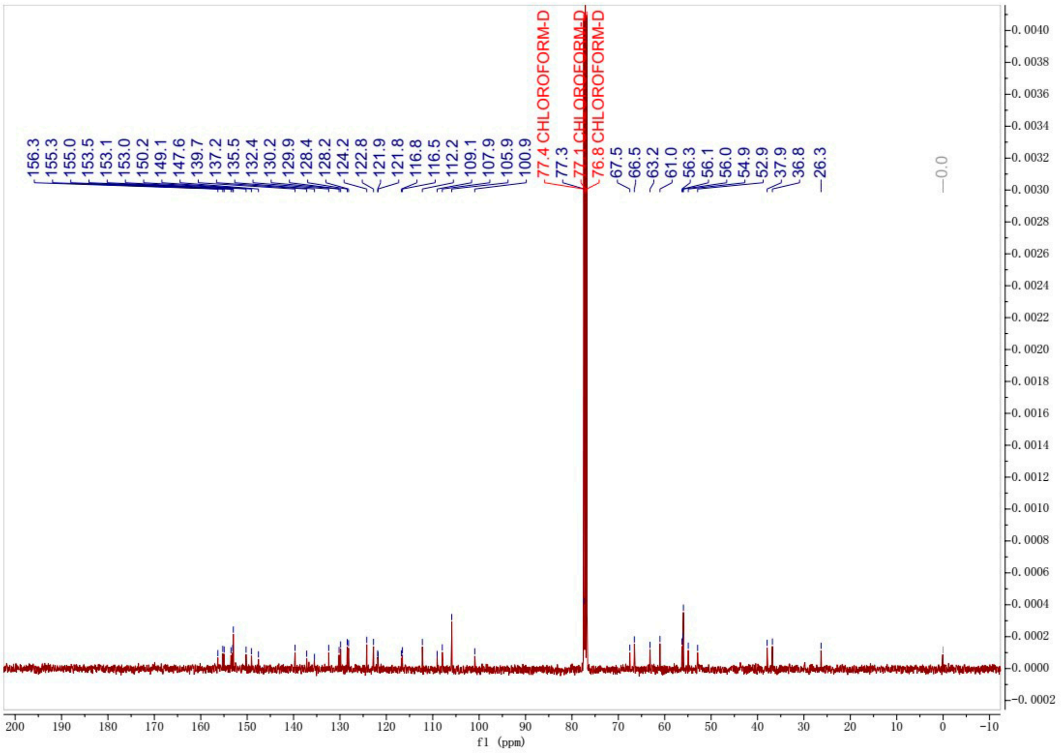

Figure S5. Magnified ^{13}C NMR spectrum

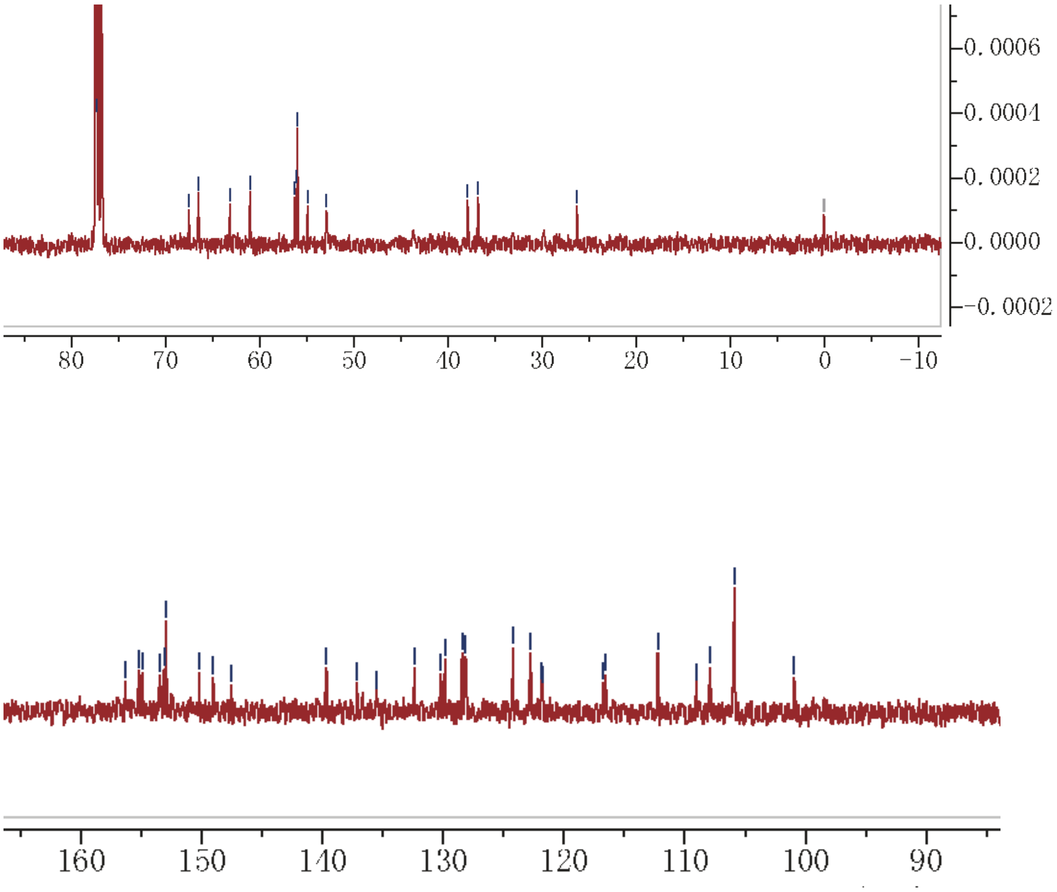

Figure S6. Magnified ^{13}C-NMR spectrum extracted from Figure S5
